# Acrylic Paints: An Atomistic View of Polymer Structure and Effects of Environmental Pollutants

**DOI:** 10.1021/acs.jpcb.1c05188

**Published:** 2021-09-15

**Authors:** Aysenur Iscen, Nancy C. Forero-Martinez, Omar Valsson, Kurt Kremer

**Affiliations:** Max Planck Institute for Polymer Research, Ackermannweg 10, 55128 Mainz, Germany

## Abstract

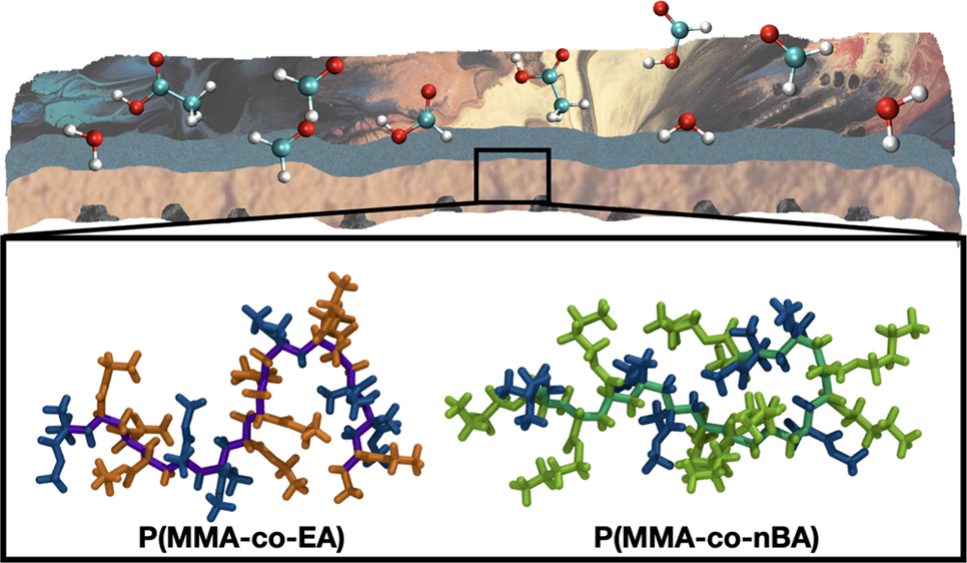

Most of the artwork and cultural heritage objects are stored in museums under conditions that are difficult to monitor. While advanced technologies aim to control and prevent the degradation of cultural heritage objects in line with preventive conservation measures, there is much to be learned in terms of the physical processes that lead to the degradation of the synthetic polymers that form the basis of acrylic paints largely used in contemporary art. In museums, stored objects are often exposed to temperature and relative humidity fluctuations as well as airborne pollutants such as volatile organic compounds (VOCs). The glass transition of acrylic paints is below room temperature; while low temperatures may cause cracking, at high temperatures the sticky surface of the paint becomes vulnerable to pollutants. Here we develop fully atomistic models to understand the structure of two types of acrylic copolymers and their interactions with VOCs and water. The structure and properties of acrylic copolymers are slighlty modified by incorporation of a monomer with a longer side chain. With favorable solvation free energies, once absorbed, VOCs and water interact with the polymer side chains to form hydrogen bonds. The cagelike structure of the polymers prevents the VOCs and water to diffuse freely below the glass transition temperature. In addition, our model forms the foundation for developing mesoscopic and continuum models that will allow us to access longer time and length scales to further our understanding of the degradation of artwork.

## Introduction

Acrylic paints have been widely used by artists since the 1900s because of their many desirable properties over traditional oils, such as fast drying times, solubility in water, and applicability to different surfaces. Although acrylic emulsion paints have long succeeded in market sales compared to other types of artists’ paints,^1^ what researchers know about acrylic paints relative to the current knowledge of oil paints in the field of conservation is limited. Therefore, there is a serious need of research to guide decisions made by conservators and scientists interested in developing better materials for preventive conservation of cultural heritage (CH). Consequently, a fundamental comprehension of structure of acrylic paints and interactions of their components is urgently needed. In this context, multiscale models are important in answering questions about degradation of synthetic polymers widely used in contemporary art.

Although acrylics are praised for many of their properties, one disadvantage of acrylics is their glass transition temperature close to room temperature. At low temperatures the paints face the danger of cracking while a temperature that is too high makes the surface of the paints sticky and more prone to collecting impurities.^2^ Changing environmental conditions, such as temperature, may lead to various structural modifications that are important in the context of acrylics degradation. While most of the degradation studies focus on the chemical modifications of the polymer chains, such as changes in surface morphology^3^ or changes of their molecular weight and solubility in organic solvents possibly due to cross-linking and oxidation,^4^ some of the physical variations due to degradation include changes in the glass transition temperature, yellowness, and surface gloss.^4^ Although acrylics are favored for their durability among other artists’ paints, they can degrade over time by either chemical degradation due to light exposure^5^ or physical degradation due to interaction with volatile organic compounds (VOCs),^6−8^ water,^9,10^ or other impurities that cause structural changes in the acrylic material. Many experimental studies in the past have focused on characterization,^11,12^ degradation,^13,14^ and conservation^15,16^ of acrylic paints in the context of conservation of cultural heritage, but not many attempts have been made in understanding the physical processes associated with structural changes that lead to degradation in acrylics. While chemical degradation commonly occurs in polymers, there might be many additional other means of degradation, such as due to disruption of polymer morphology.^17^ Recently, Yang et al. studied diffusion of methane and carbon dioxide in poly(acrylates) by using an atomistic model. They claim that the side chain length of acrylics plays an important role in intermolecular interactions and the shielding of the polar ester group is responsible for the decrease in glass transition temperature with increasing side chain length.^18^ In another study, molecular dynamics simulations were employed to examine the mechanism of water recrystallization in poly(methyl acrylates) by determining the relationship between glass transition temperature of polymers and water diffusion.^19^

In this study, we develop a computational model to focus on how VOCs and water in the environment interact with the acrylic polymers found in modern paints. There are numerous components that make up acrylic paints that give this quick-drying, easy to use, versatile, water-based paint the qualities that make them so popular in the art world. Out of the three major components—pigment, binder, and vehicle (water)—acrylic binders are responsible for the overall quality of the paint, while the pigment and vehicle determine the quality of the color and the ease of application. Because most of the artworks are indeed composed of different layers that make it impossible to directly apply molecule based computational models, we turn our attention to the binder materials since it makes up most of the painting and the binder structure is crucial to understanding degradation, cracking, etc., in response to changes in environmental conditions, such as temperature, relative humidity, and accumulation of VOCs, over time. Because experimental data concerning structure of acrylic binders are scarce, we first test our model against known properties of the individual components of the copolymers found in acrylic binders, which are poly(methyl methacrylate) (PMMA), poly(ethyl acrylate) (PEA), and poly(*n*-butyl acrylate) (P(*n*BA)). For the acrylic paints, we consider two different types of acrylics binders, P(MMA-*co*-EA) and P(MMA-*co*-nBA), that were popular amount artists and compare their structure by calculating properties such as glass transition temperature, diffusion coefficients, and small-angle X-ray scattering spectra. Finally, we present some of our findings regarding interaction of acrylic polymers and pollutants, such as VOCs or water. Furthermore, this microscopic investigation into acrylics structure will provide us ways to develop other multiscale models in the coarse-grained and continuum level that can take into account different environmental factors and let us study longer time scales relevant to degradation. Other than the more direct outcomes of this study in the conservation of cultural heritage, the model developed can also be adapted to study other processes since acrylic polymers are widely employed in many industries,^20^ and understanding polymer degradation and lifetime with data-based methods has been proven beneficial to address more pressing global environmental concerns.^21^

## Methods

### Simulations of Homopolymers

We performed molecular dynamics (MD) simulations of bulk homopolymers (PMMA, PEA, and P(*n*BA)). The isotactic 15-mer polymer chains were constructed with AmberTools^22^ and placed randomly in a box with initial dimensions 9 × 9 × 9 nm^3^ for 100 15-mer homopolymer chains by using Packmol.^23^ We chose a 15-mer polymer chain because while it is much lower than the molecular weight of polymers used in acrylic paints and experiments, it is computationally feasible to study large systems at long times. Because we are way below the entanglement lengths (*M*_e_ of PMMA = 12,500 g/mol, *M*_e_ of PEA = 7770 g/mol^24^), we can study the Rouse behavior of these polymers from our simulations. Simulations of homopolymers were performed by using the general Amber force field (GAFF)^25^ and modified Optimized Potentials for Liquid Simulations force field (OPLS) based on our earlier work^26^ to compare the two force fields and validate our model. The MD simulations were performed with the Gromacs 2019.4^27,28^ software. Bonds involving hydrogen atoms were constrained by using the LINear Constraint Solver (LINCS) algorithm.^29^ The Verlet cutoff scheme^30^ was used for neighbor searching. Long-range electrostatics were determined with the smooth particle mesh Ewald (PME)^31^ method using cubic interpolation and a Fourier grid spacing of 0.16 nm. A cutoff of 1 nm was used for evaluation of all nonbonded interactions. Atomic coordinates were saved every 100 ps for the trajectory analysis. Each system was minimized for 1000 steps by using the steepest descent algorithm. Following the minimization, we equilibrated for 200 ps using the NVT (constant number of particles, volume, and temperature) ensemble using the v-rescale coupling method^32^ at 600 K with a 2 fs time step. We further equilibrated the system with NPT (constant number of particles, pressure, and temperature) ensemble for 10 ns using the Nosé–Hoover thermostat^33,34^ and Parrinello–Rahman barostat^35,36^ to maintain temperature (600 K) and pressure (1 bar), respectively, to make sure the systems were fully equilibrated and the volume of the box was converged. After this short equilibration at 600 K, the temperature was decreased to 100 K by using a cooling rate of 1.2 × 10^12^ K/min or 20 K/ns to calculate the glass transition temperature and other temperature-dependent properties. The coordinates of the system at temperature intervals of 50 K were saved and further equilibrated for 10 ns by using NPT ensemble to study the temperature-dependent properties of the paint.

### Simulations of Copolymers

#### Bulk Simulations

We further performed simulations of copolymer chains made up of 40% MMA and 60% EA (or *n*BA). This ratio of polymers in the copolymer was based on experimentally determined compositions of binders used in acrylic paints, Rhoplex (Primal) AC-33 and AC-235 by Rohm & Haas.^37−39^ To introduce some randomness into our copolymers, we modeled five different copolymer chains with the same composition by only changing the order of the monomer sequences. The monomers were distributed randomly in each chain. The simulations were set up in the same way as above by using 100 15-mer chains (20 of each copolymer sequence). For all copolymer simulations, we modeled our system with GAFF based on the results obtained from homopolymer simulations. Simulation protocol including annealing was performed in the same way. Systems were equilibrated for 100 ns at each temperature (100–600 K with 50 K intervals) by using the NPT ensemble before analysis. For the copolymer simulations in the bulk phase, the initial box size was 9 × 9 × 9 nm^3^.

Finally, we incorporated volatile organic compounds (VOCs) to our copolymer simulations to study their interaction with the polymer matrix. The force field parameters for acetic acid, formic acid, and formaldehyde were previously developed by van der Spoel and co-workers^40,41^ according to GAFF. In the bulk copolymer simulations, we used 1000, 3000, and 6000 ppm concentration of each VOC. This means we had 3 acetic acid, 3 formic acid, and 6 formaldehyde for 1000 ppm, 9 acetic acid, 12 formic acid, and 18 formaldehyde for 3000 ppm, and 18 acetic acid, 24 formic acid, and 36 formaldehyde molecules for 6000 ppm according to our box size. After randomly inserting VOCs in the bulk copolymers equilibrated at 600 K using Packmol, we used the same annealing procedure as before to obtain correct densities of copolymer systems with the VOCs embedded in the bulk phase. These systems were further equilibrated for 30 ns at each temperature (250–500 K with 50 K intervals) by using the NPT ensemble.

### Solvation Free Energy Calculation

The solvation free energies of VOCs in the copolymers were calculated by using the thermodynamic integration (TI) method. For these calculations, a smaller system consisting of 10 copolymer chains and 1 VOC molecule (acetic acid, formic acid, or formaldehyde) in a cubic box of initial size 3.5 × 3.5 × 3.5 nm^3^ was used. For each VOC in each copolymer (P(MMA-*co*-EA) and P(MMA-*co*-*n*BA)), we calculated the solvation free energy by first decoupling the electrostatic interaction using 21 simulations in steps of 0.05 (λ_C_ = 0, 0.05, ..., 0.95, 1.0), followed by decoupling the Lennard-Jones interaction busing 41 simulations with uneven values of λ_LJ_ as suggested by other studies.^42^ The following 41 values of λ_LJ_ were used: 0.00, 0.10, 0.20, 0.22, 0.24, 0.26, 0.28, 0.30, 0.32, 0.34, 0.36, 0.38, 0.40, 0.41, 0.42, 0.43, 0.44, 0.45, 0.46, 0.47, 0.48, 0.49, 0.50, 0.52, 0.54, 0.56, 0.58, 0.61, 0.64, 0.67, 0.70, 0.73, 0.76, 0.79, 0.82, 0.85, 0.88, 0.91, 0.94, 0.97, and 1.00. For each λ_C_ and λ_LJ_, the initial coordinates were minimized for 5000 steps by using the steepest descent algorithm, followed by a 200 ps NVT simulation at 298 K by using the v-rescale coupling method. Another equilibration was performed in the NPT ensemble for 200 ps by using the same settings as before. After the equilibration, a production run of 1 ns was run for each of the λ steps. The free energy difference between fully coupled and fully uncoupled states was calculated by using the Bennet Acceptance Ratio (BAR) implemented in Gromacs.^43^

### Trajectory Analysis

For the calculation of diffusion coefficients, we ran an extra 10 ns of simulations in NVT for all systems due to possible error associated with MSD calculation in NPT ensemble.^44^ We calculated the diffusion coefficients based on the center of mass of monomers in the polymer chains and the center of mass of molecules for the VOCs. The diffusion coefficients were calculated by plotting MSD/time vs 1/time and extrapolating to find the limit at *t* → *∞* due to limited sampling and short simulation time. Without linear extrapolation to *t* → *∞*, the actual value of MSD/time in the still subdiffusive regime at this time gives a value that is too large, and therefore the calculated diffusion coefficients are overestimated. On the other hand, linear extrapolation typically underestimates the diffusion coefficients. To account for this we use time increments of different lengths (e.g., 10, 20, and 50 ns) in our analysis and extrapolate the apparent diffusion constants. Because sampling issues become critical at low temperatures (*T* < 300 K), we expect that the extrapolation method used here will minimize the inaccuracies associated with estimating diffusion coefficients at short simulation times. We calculated the glass transition temperatures by fitting the data to two piecewise linear functions, using the pwlf package.^45^ All snapshots from simulations were rendered by using the Visual Molecular Dynamics (VMD) software.^46^ For hydrogen bond analysis, the criteria of hydrogen–donor–acceptor angle of 30° and a donor–acceptor distance of 0.35 nm were used. The solvent accessible surface area was calculated by using a probe radius of 0.14 nm. The intermediate scattering functions were calculated by using LiquidLib code.^47^

## Results and Discussion

### Atomistic Model and Force Field Validation

**Figure 1:**
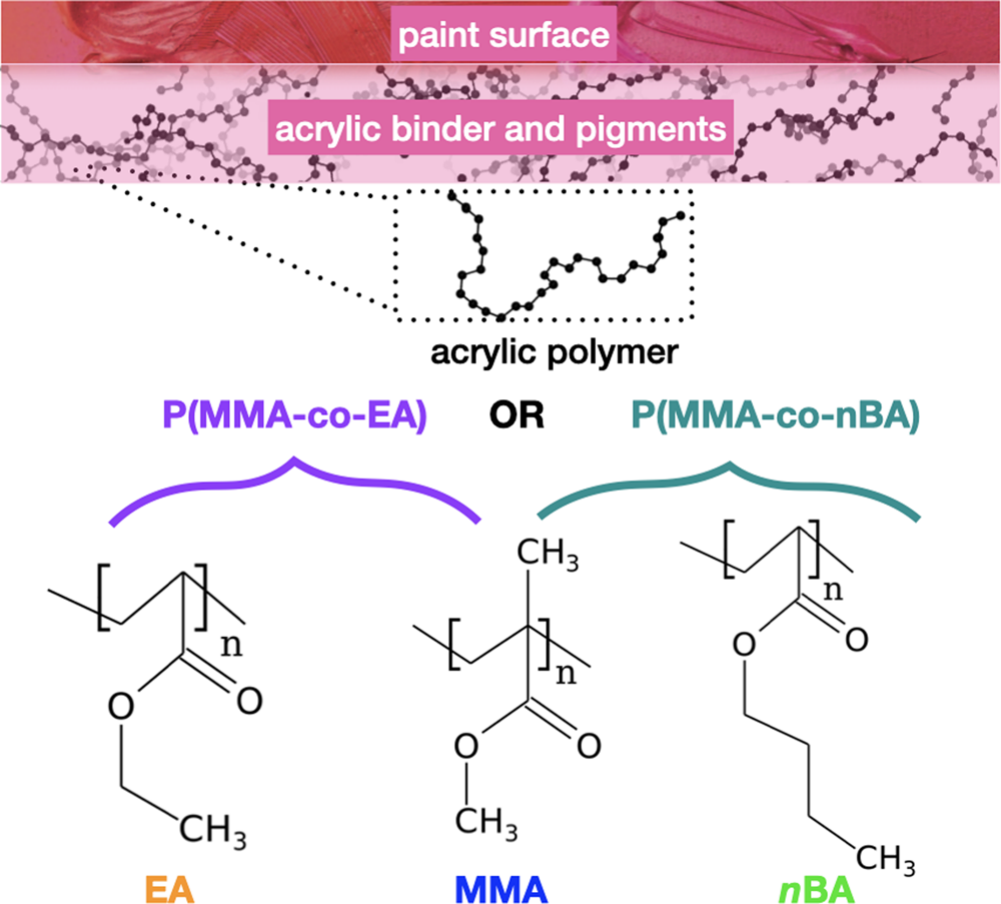
Acrylic paints are made up of copolymer binders composed of the three monomers methyl methacrylate (MMA, C_5_O_2_H_8_), ethyl acrylate (EA, C_5_O_2_H_8_) and *n*-butyl acrylate (*n*BA, C_7_H_12_O_2_).

Acrylic paint formulations vary significantly depending on the choice of additives, pigments, surfactants, etc., by the manufacturers and artists. While it is difficult to assess the composition of each paint, model acrylic latex paints contain about 41% water, 32% polymer binder, and 6.5% pigments plus additives.^48^ During the drying process, water evaporates from the surface, which results in a polymer fraction of about 60–70 vol %.^49^ While early paints consisted of copolymers of PMMA and PEA, the composition of the binder changed to copolymers of PMMA and P(*n*BA) in the late 1980s (Figure 1). Because of the scarcity of experimental data on these acrylics, we first modeled the components separately to test our model against some of the known properties for these polymers, such as the glass transition temperature (*T*_g_), self-diffusion coefficients, and small-angle X-ray scattering (SAXS) spectra. To most accurately model the polymeric binder material, we tested two different molecular force fields, GAFF^25^ and modified OPLS based on our earlier work^26^ and performed molecular dynamics simulations.

**Figure 2:**
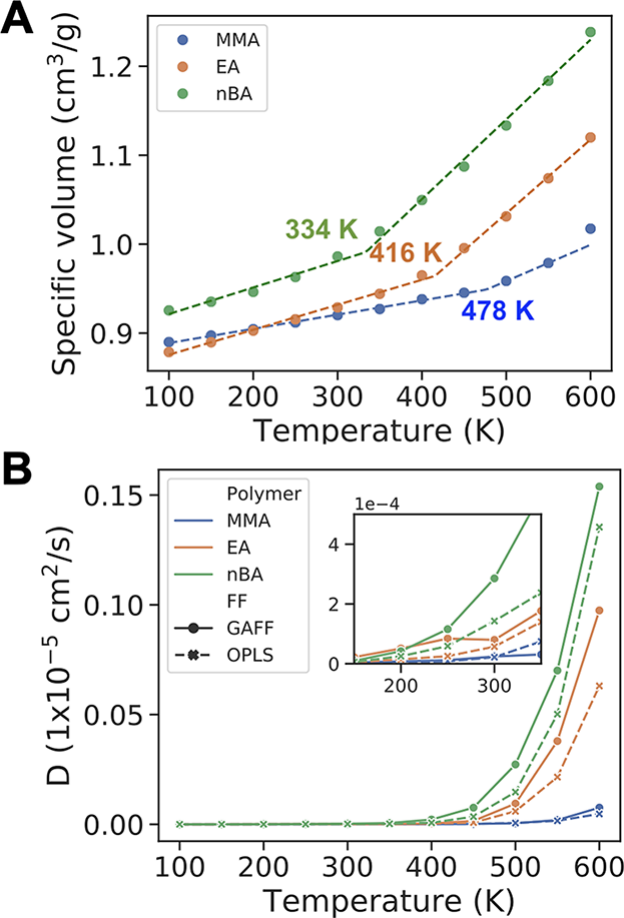
(A) Specific volume and glass transition temperatures and (B) self-diffusion coefficients of PMMA, PEA, and P(*n*BA) calculated by using GAFF (solid lines) and OPLS (dashed lines). Figure S2 shows log(*D*) plotted vs temperature. Figure S3 shows the mean-square displacement data used to calculate the diffusion coefficients.

Most of the preventive measures in conservation of artwork focus on developing tools to maintain an appropriate environment for the paintings. This can be tricky for acrylic paintings since the glass transition temperatures for acrylic binders (P(MMA-*co*-EA) and P(MMA-*co*-*n*BA)) are designed to result in a *T*_g_ that is near or below room temperature to avoid cracking in the paintings at low temperatures.^50,51^ However, their low *T*_g_ makes them rubbery at room temperature, increasing their affinity for attracting dirt and airborne pollution by sticking to the surface at ambient temperatures.^2^ From a computational perspective, it is rather difficult to calculate *T*_g_ due to inaccessibility of long time scales in MD simulations required for experimentally relevant cooling rates. Moreover, there are several different ways of calculating *T*_g_, such as considering the changes in the specific volume or in different components of bonded and nonbonded energies.^52^ In Figure 2, we show results from the common practice of calculating specific volume from the density of the simulation box, which is a good approximation in most cases.^19,53−55^ The average densities of PMMA, PEA, and P(*n*BA) decrease with increasing temperature. As a comparison, the average calculated densities of PMMA, PEA, and P(*n*BA) at 300 K are 1.087, 1.076, and 1.014 g/cm^3^, respectively, where a longer side chain of polymer results in a lower density. The density values from our simulations are in good agreement with experimental densities of 1.17,^56^ 1.09,^57^ and 1.04^57^ g/cm^3^ for PMMA, PEA, and P(*n*BA), considering our simulations are of shorter (15-mer) chain lengths, which have a lower density compared to those used in experiments due to free volume around the chain ends. The glass transition temperatures calculated by using the specific volume in Figure 2 for the three different homopolymers are 478 K for PMMA, 416 K for PEA, and 334 K for P(*n*BA) with GAFF. In comparison, with the OPLS force field (Figure S1) the calculated *T*_g_ is 480 K for PMMA, 425 K for PEA, and 385 K for P(*n*BA). The variation of *T*_g_ from difference force fields results from small changes in density, structure, and packing of polymer chains due to differences in parameters and becomes more pronounced as the side chain of the polymer becomes longer. While these *T*_g_ values are higher compared to those measured in experiments, i.e., 333–387 K^53,58−60^ for PMMA, 249 K^61^ or 231 K^62^ for PEA, and 223 K^59^ for P(*n*BA), there is good qualitative agreement between our simulations and the experimental data where we observe a decrease in the *T*_g_ with growing length of polymer side chains. This trend of decreasing *T*_g_ with increasing side chain length is often discussed in terms of the “plasticization effect”,^63,64^ but in our case the side groups (methyl, ethyl, and butyl) are quite short to decide whether it is a packing or plasticization effect. The differences in MD-calculated *T*_g_ and experimental *T*_g_ is a result of fast cooling rates (1.2 × 10^12^ K/min) and limitations in system size.^18^ Others have proposed an adjustment to the *T*_g_ values using the Williams–Landel–Ferry (WLF) equation for linking the experimental and simulation cooling rates.^19,65,66^
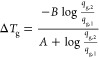
1where Δ*T*_g_ = *T*_g,1_ – *T*_g,2_ and *q* is the cooling rate from simulations (1) and experiments (2). For our case, we used the values of *A* (17.7 K) and *B* (59.3 K) derived from simulation and experimental data using PMMA.^65^ For PEA and P(nBA) similar fitting parameters are not available; thus, we used the PMMA parameters to provide a first comparison. The experimental cooling rate is 10 K/min, and the computational cooling rate is 1.2 × 10^12^ K/min. This results in Δ*T*_g_ = 99.2 K. With this adjustment the calculated *T*_g_ becomes 379, 317, and 235 K for PMMA, PEA, and P(*n*BA), respectively. The WLF adjustment to the simulation *T*_g_ results in perfect agreement with experimental *T*_g_ for PMMA (333–387 K^53,58−60^) when differences in cooling rates are taken into account. Because *A* and *B* values used in eq 1 for PEA and P(nBA) are not readily available, the estimation using the data for PMMA does not produce as good of an agreement with experiments (249 K^61^ and 231 K^62^ for PEA and 223 K^59^ for P(*n*BA)). However, the qualitative trends regarding *T*_g_ and side-chain length observed in experiments are preserved in our simulations.

The glass transition temperature is negatively correlated to self-diffusion coefficients of the polymer chains. For all three polymers studied, the self-diffusion coefficients are larger when GAFF is used rather than OPLS (Figure 2B), and the diffusivity of the polymer is in the order P(*n*BA) > PEA > PMMA for both force fields. The larger the side chain is for these polymers, the faster the polymer chains diffuse in the bulk polymer phase. There is very little diffusion at low temperatures (<500 K) below the limit that can be detected by the current simulations. Experiments show the diffusion of PMMA is ∼10^–5^ cm^2^/s for low molecular weight chains,^67^ which is consistent with our simulations considering the differences in size of the polymer chains.

**Figure 3:**
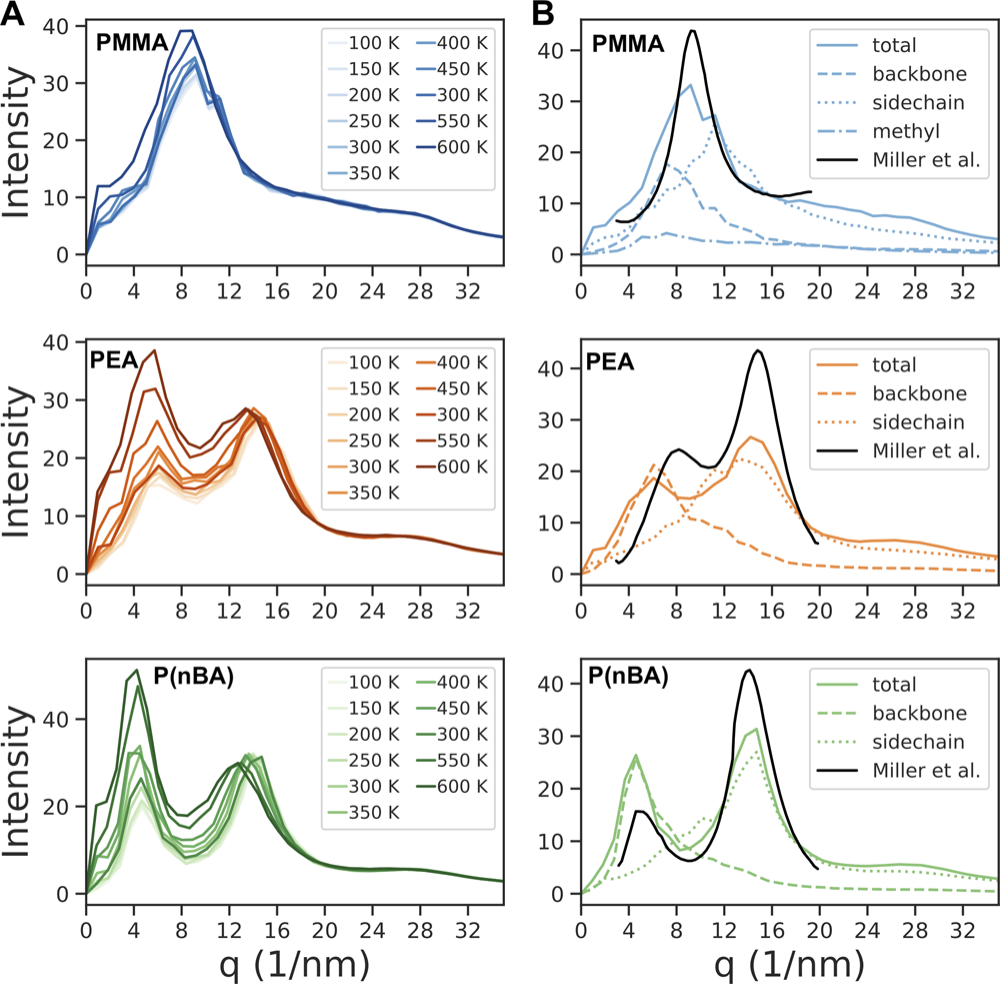
Small-angle X-ray scattering structure factor (A) as a function of temperature and (B) at 300 K of PMMA, PEA, and P(*n*BA) calculated via GAFF. The experimental data at 298 K from Miller et al.^69^ are shown for comparison.

Finally, we also calculated the small-angle X-ray scattering structure factor to investigate how well these force fields can reproduce the structural properties of these homopolymers. In Figure 3, our calculated SAXS structure factor for PMMA, PEA, and P(*n*BA) match perfectly with those measured in experiments at room temperature.^68,69^ While the SAXS structure factor of PMMA consists of a single peak (*q* = 9 nm^–1^), PEA (*q* = 6 nm^–1^ and *q* = 14 nm^–1^) and P(*n*BA) (*q* = 4 nm^–1^ and *q* = 14 nm^–1^) show two separate peaks. The relative intensity of the peaks changes as the temperature increases, which was previously observed in polystyrene SAXS spectra.^70,71^ Considering the significant differences between PMMA vs PEA and P(*n*BA), we investigated the origin of these peaks found in the structure factor. As it can be seen in Figure 3B, backbone and side-chain atoms are responsible for the different peaks that appear in the SAXS structure factor. Here the peak position is most relevant, and the amplitudes are not simply additive. The first peak in the structure factor (*q* ∼ 4 nm^–1^) results from the backbone atoms of the polymers. The average distance between the atoms is roughly 2π/*q*, which means the backbone–backbone distance increases from ∼8 Å for PMMA to ∼16 Å for P(nBA) as a result of repulsive interactions from longer side chains that prevent the backbone atoms from approaching closer to each other. The length of the side chain also significantly impacts the position of the second peak at a higher scattering angle. While for the polymer with the shortest side chain, e.g., PMMA, this peak is merged together with the backbone peak and disappears in the total structure factor, the polymers with the longer side chains show two distinct peaks. Knowing where the peaks in the structure factor come from, we can further understand the effect of temperature. The intensity and width of the peaks are related to the frequency of occurrence of that distance. At higher temperatures, there is more room for the chains to move, diffuse, bend, coil, expand, etc. The amorphous halo around 14–16 nm^–1^ with corresponding distance of about 4–5 Å becomes significantly broader and less structured with increasing temperature as we expected. With increasing temperature the side chains become more coil-like, which obviously leads to a stronger chain–chain excluded volume effect and the increase in the intensity of the peak at ∼4 nm^–1^, which corresponds to a distance of about 16 Å.

The results from our homopolymer simulations show good agreement in terms of the trends for both force fields. The diffusion in GAFF is slightly larger compared to that in OPLS. The relative differences in glass transition temperature are reasonably reproduced compared to experimental values. It is important to mention here that the glass transition temperature measured in experiments also is dependent on the method of measurement, data analysis technique, and cooling rates. Therefore, we believe that our calculated *T*_g_ values are consistent with experiments considering previously mentioned limitations of MD simulations. Finally, calculated structure factors from both GAFF and OPLS show good agreement with experiments although the GAFF structure is better at capturing the peaks, especially for PMMA and PEA. While both of these force fields have shown good compatibility to study this problem, we have chosen GAFF for modeling the copolymers found in acrylic paints because this force field seems to match experiments slightly better.

### Properties of Acrylics Found in Paints

Deterioration of acrylics takes place over years and is a result of internal processes and of external environmental factors,^4^ such as light,^5^ migration of surfactants,^72^ and temperature as well as relative humidity.^37,50^ Through a combination of physical and chemical processes, aging of modern paints starts from the surface and advances to the inner layers of the paint, where changes in the structure of the polymeric binder cause defects that facilitate the diffusion of small particles.^3^

**Figure 4:**
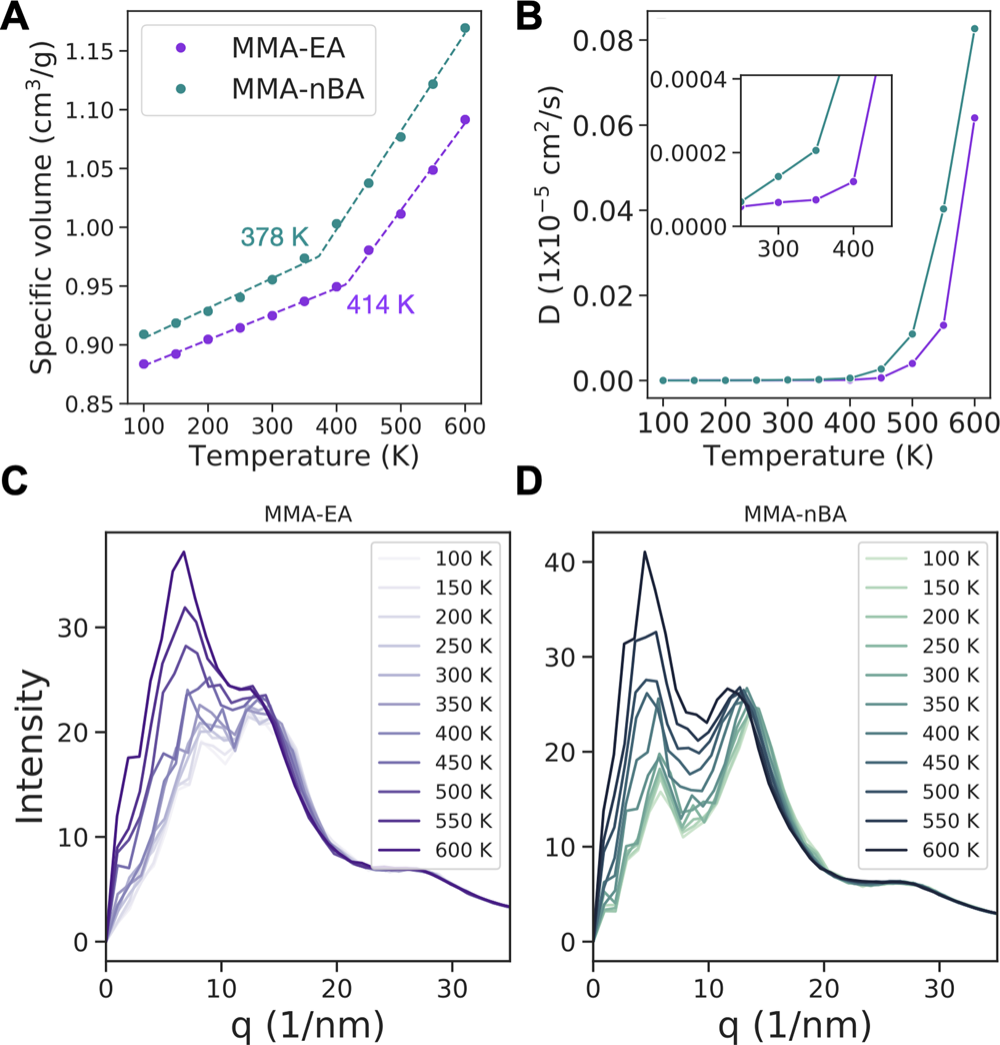
(A) Specific volume and glass transition temperatures, (B) self-diffusion coefficients. and (C, D) small-angle X-ray scattering structure factors of P(MMA-*co*-EA) and P(MMA-*co*-*n*BA) at different temperatures. Figure S5 shows log(*D*) plotted vs temperature. Figure S5 shows the mean-square displacement data used to calculate the diffusion coefficients.

In an effort to improve the quality of acrylic paints, in the 1950s, the manufacturers of these paints switched from using P(MMA-*co*-EA) binders to a P(MMA-*co*-*n*BA) binder,^2^ with measured *T*_g_ of 289 K^73^ and 280 K,^74,75^ respectively. Our simulations show that the *T*_g_ of P(MMA-*co*-*n*BA) (378 K) is smaller than the *T*_g_ of P(MMA-*co*-EA) (414 K) (Figure 4A). We calculated the diffusion coefficients of the polymer chains by using the center of mass of each chain using an extrapolation method (see the Methods section). The diffusion coefficient of the polymers is on the order of 10^–9^ cm^2^/s at 300 K and increases significantly at 600 K. Here we also note the P(MMA-*co*-*n*BA) binder is more mobile than P(MMA-*co*-EA), in agreement with the homopolymer diffusivities calculated in the previous section.

Perhaps one of the most significant differences in the structure of the two acrylic binders is obvious when we examine the SAXS data (Figure 4C,D), where P(MMA-*co*-*n*BA) shows two distinct peaks similar to P(*n*BA) throughout the temperature range studied while the peaks in P(MMA-*co*-EA) structure are less distinguishable due to the small side chain of the PEA component (Figure S6). Further investigation of our simulations reveals that these copolymers expand appreciably with increasing temperature. This decrease in density results in empty spaces, or “cages”, between the copolymer chains. Formation of these cages in the polymer may play a role in the overall change in the structure of the material and absorption of molecules, such as VOCs or water, from the atmosphere. In Figure S7, we can see the increase in solvent accessible surface area (SASA) with temperature for both copolymers. Furthermore, in comparing P(MMA-*co*-EA) and P(MMA-*co*-*n*BA), we notice that the latter has a larger accessible surface area, which is due to larger side chains of the *n*BA monomer. Therefore, while the longer side chain of P(MMA-*co*-*n*BA) lowers the *T*_g_, it also allows for a more porous material that may not be a desirable property from a structural point of view for limiting absorption and diffusion of VOCs.

**Figure 5:**
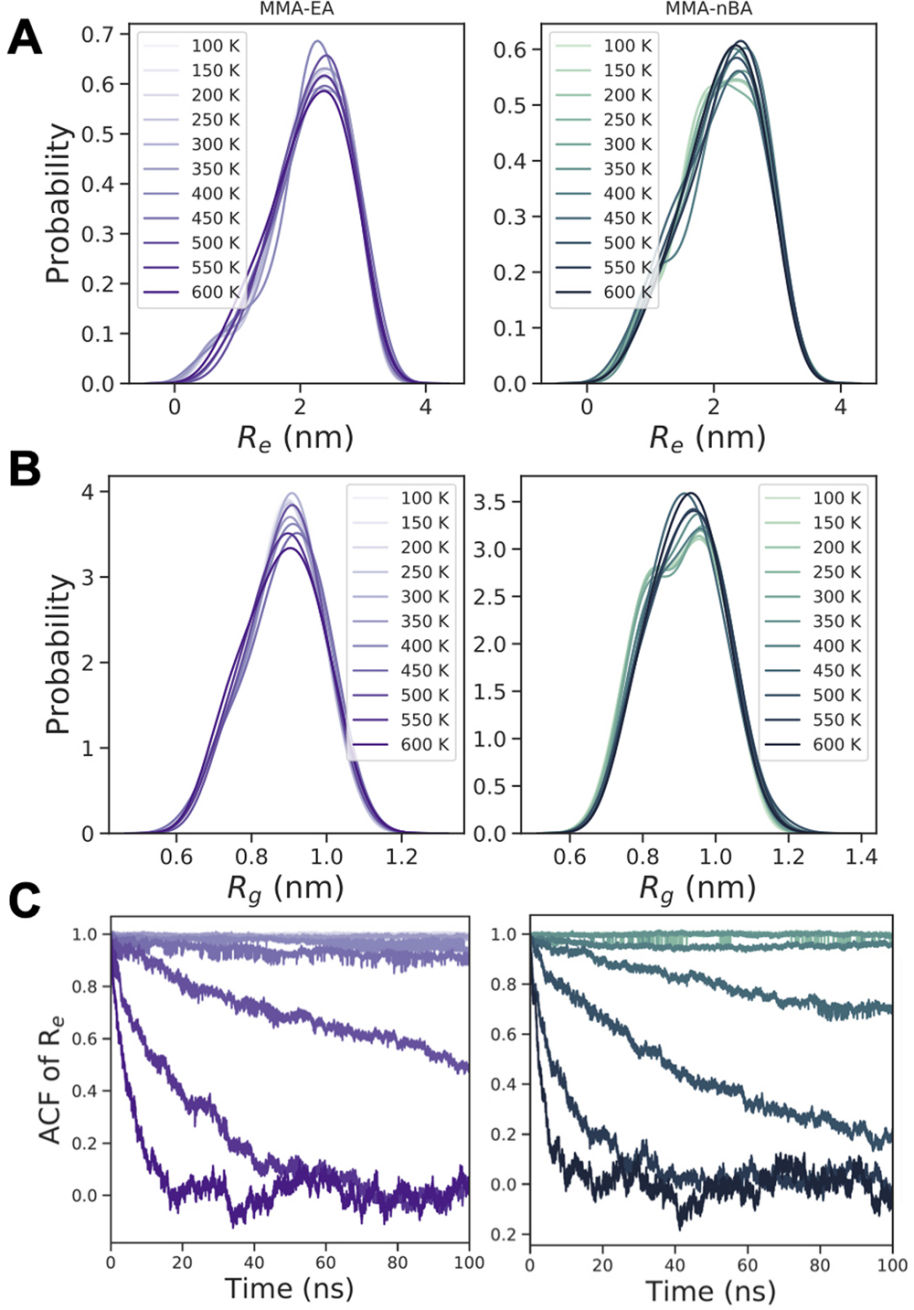
Probability distribution of (A) end-to-end distance, *R*_e_, and (B) radius of gyration, *R*_g_, from the last 10 ns of trajectories and (C) end-to-end vector autocorrelation function for P(MMA-*co*-EA) and P(MMA-*co*-nBA) in bulk simulations at different temperatures.

Furthermore, we examined other statistical properties of the polymer chains, such as end-to-end distance (*R*_e_) and radius of gyration (*R*_g_). The probability distributions of the *R*_e_ and *R*_g_ are shown in Figure 5, where we observe a marginal increase in both values with increasing temperature. The structural changes that occur around the glass transition temperature are also evident in the end-to-end vector autocorrelation functions shown in Figure 5C. While above *T*_g_ the end-to-end vector autocorrelation functions decay to zero over the simulation time, this is not true in the glassy state, indicating slow or inhibited relaxation of the polymer chains.

The results obtained from our copolymer simulations can consistently reproduce the trends observed in the homopolymer simulations in terms of the calculated properties, such as glass transition temperature, diffusion coefficients, and structure factors. When PMMA is copolymerized with softer PEA or P(nBA) monomers, the result is a more easily manageable material at room temperature with a lower glass transition temperature. Furthermore, the two choices of acrylic binders P(MMA-*co*-EA) and P(MMA-*co*-*n*BA) show slight structural variations arising from the different side-chain lengths. Therefore, even though directly comparable experimental data concerning acrylic binders are few, based on overall consistency in our simulations and good agreement with available data, we believe our models show promise in capturing the structural details of acrylics at the microscopic level.

### Effects of VOCs and Water on Acrylic Structure

One of the main long-term objectives of this study is to determine the threshold region of concentration of pollutants that triggers significant degradation of artwork. Eventually we are interested in understanding the action of these pollutants inside the matrix of the art material and how the variations in their local density can lead to microscopic stresses in a material leading to e.g. brittle failure. Volatile organic compounds can be emitted by the packaging material or the artwork materials other than the paint. When emitted by the packaging material, some of these compounds may be absorbed by the artwork and cause variations in its structure that can lead to degradation. Some of the VOCs most commonly emitted are acetic acid, formic acid, and formaldehyde. Acrylics can also come into contact with water as a result of increase in the relative humidity of the room or storage enclosures. In this section, we will look at the interaction of VOCs and water with the acrylic binder materials in bulk.

### Free Energy of Solvation

Why do VOCs and water get absorbed into the acrylic paints at all? As part of the film formation process, water evaporates from the acrylic emulsions to bring the polymers together during drying. However, when this happens it has been shown experimentally that pores or voids are left in the film resulting from imperfect particle packing.^3^ These defects allow particles such as VOCs and water to become absorbed and trapped in the film, which may have consequences in degradation. It is a challenge to tackle the question of degradation, which happens over long times in acrylics, by using state of the art molecular simulations at the atomistic level. However, understanding the specific interactions between molecules is essential in calculating properties such as solvation free energies and diffusion that will contribute to developing a mesoscopic model in the continuation of this study.

**Figure 6:**
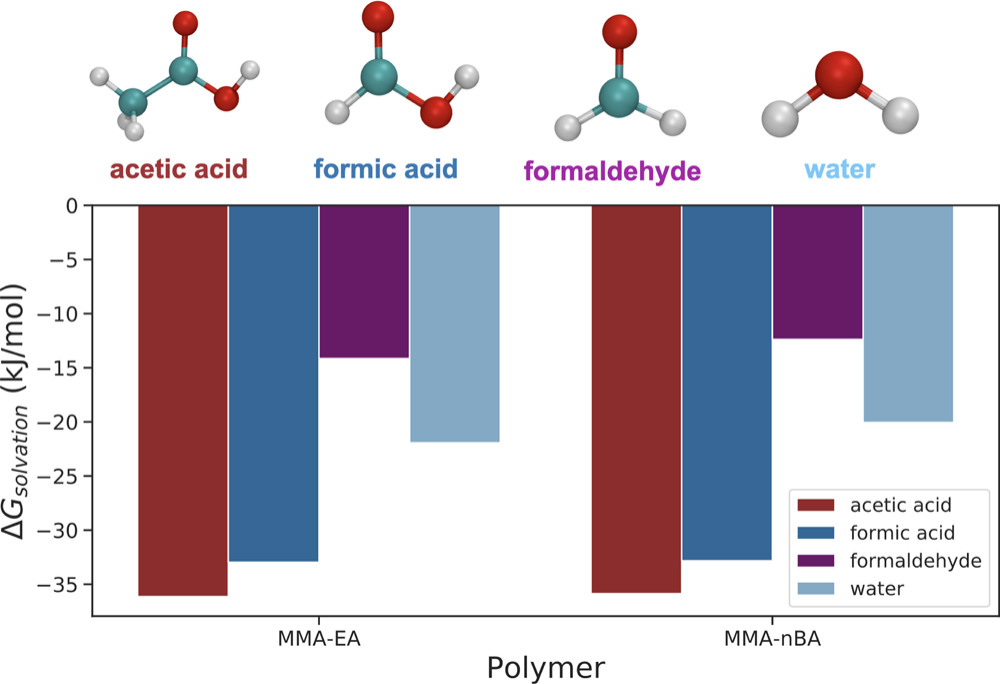
Different volatile organic compounds used in this study and their free energy of solvation in P(MMA-*co*-EA) and P(MMA-*co*-*n*BA) at 298 K.

The solvation free energies of VOCs and water in P(MMA-*co*-EA) and P(MMA-*co*-*n*BA) were calculated by using thermodynamic integration (see the Methods section for details). The solvation free energies reported in Table 1 and shown in Figure 6 indicate that the solvation behavior of VOCs is very similar in both copolymers, with slightly more favorable solvation in P(MMA-*co*-EA). When the VOCs are compared to each other, the species with carboxylic acid groups, i.e., acetic acid and formic acid, prefer to be solvated more than formaldehyde. Water solvation free energy, on the other hand, falls between formic acid and formaldehyde. At a first glance, this trend is not intuitive since we would expect the relative hydrophobicities of the pollutants (formaldehyde > acetic acid > formic acid > water) to be reflected in the calculated solvation energies. Nevertheless, the observed trend is better understood when we discuss intermolecular interactions, such as hydrogen bonding, in later sections.

**Table 1:** Solvation Energies Calculated for Acetic Acid, Formic Acid, Formaldehyde, and Water in P(MMA-*co*-EA) and P(MMA-*co*-*n*BA) (All Values in kJ/mol)

	P(MMA-*co*-EA)			P(MMA-*co*-nBA)		
VOC	Coulombic	van der Waals	total	Coulombic	van der Waals	total
acetic acid	–18.25 ± 0.15	–17.89 ± 0.32	–36.14	–17.91 ± 0.25	–17.95 ± 0.38	–35.86
formic acid	–20.95 ± 0.45	–12.01 ± 0.19	–32.96	–20.91 ± 0.07	–11.92 ± 0.27	–32.83
formaldehyde	–7.69 ± 0.17	–6.46 ± 0.16	–14.15	–6.63 ± 0.12	–5.75 ± 0.12	–12.38
water	–22.73 ± 0.55	0.80 ± 0.18	–21.93	–22.06 ± 0.33	2.01 ± 0.24	–20.05

### Diffusion Mechanisms

**Figure 7:**
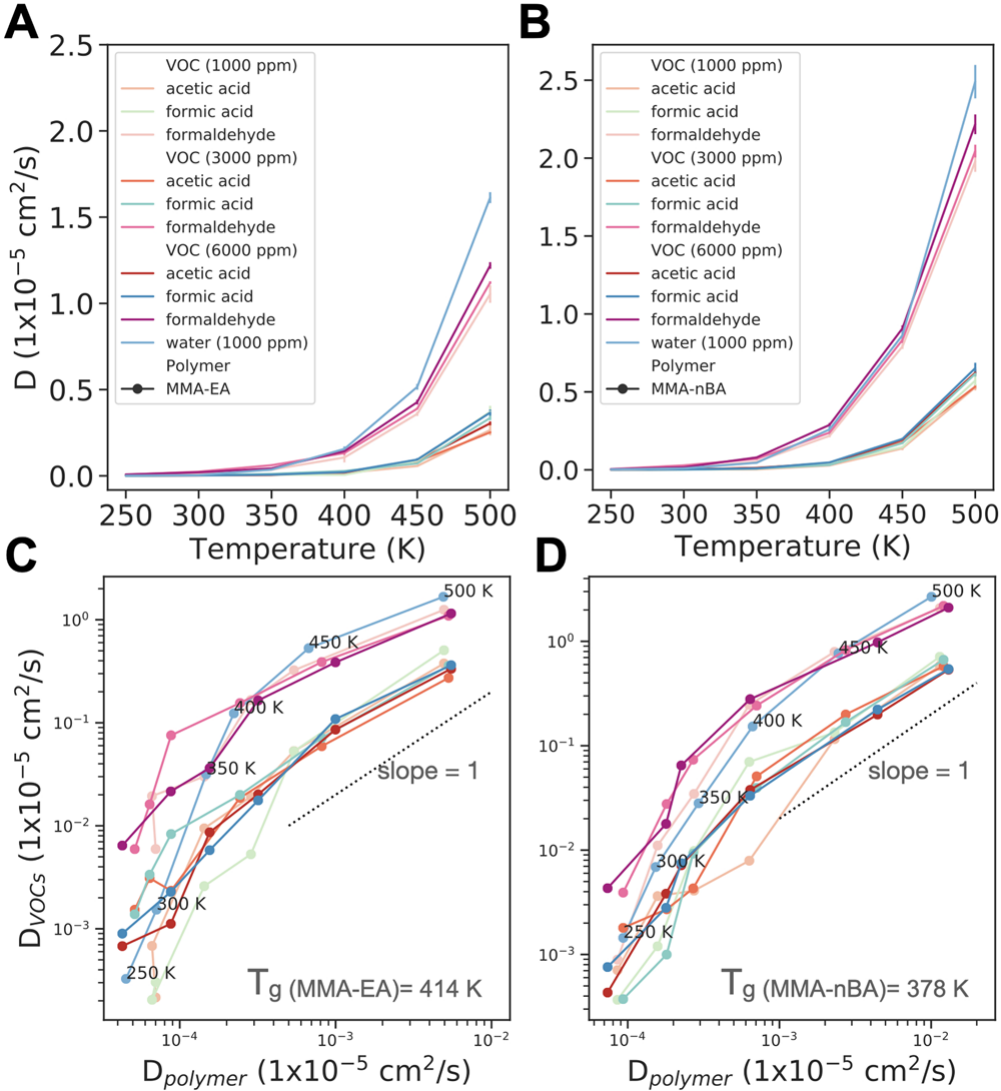
Self-diffusion coefficients of acetic acid, formic acid, formaldehyde, and water in (A) P(MMA-*co*-EA) and (B) P(MMA-*co*-*n*BA) as a function of temperature. Figure S8 shows log(*D*) plotted vs temperature. The average *D* values from five 10 ns trajectories are plotted with error bars referring to standard error of mean. See Figures S9–S12 for mean-square displacement used to calculate the *D*. Relationship between the diffusion coefficients of VOCs/water and polymer chains for (C) P(MMA-*co*-EA) and (D) P(MMA-*co*-nBA). Diffusion coefficients increase with temperature. Temperature labels are shown for water diffusivities in (C) and (D) to aid the eye.

What is even more interesting is what happens to the VOCs once they are absorbed into the acrylic paints as a result of this favorable interaction. We included VOCs in our bulk copolymers at concentrations of 1000, 3000, and 6000 ppm. We recognize that these concentrations are relatively large compared to experimentally measured concentrations of these VOCs in acrylics,^6^ which is a drawback of atomistic simulations due to limitations in system size. We have also tested the effect of water absorption by performing a separate simulation with ∼1000 ppm water concentration. Quantitatively, in Figure 7 we can observe that the VOC diffusion is correlated to the solvation free energies and follows the order acetic acid < formic acid < formaldehyde. Our results show that the diffusion of VOCs are on the order of 10^–8^–10^–7^ cm^2^/s at 300 K and increase to ∼10^–5^ at higher temperatures. For example, the calculated diffusion coefficient of formaldehyde is 1.94 × 10^–7^ cm^2^/s at 300 K and 3.01 × 10^–7^ cm^2^/s at 350 K for P(MMA-*co*-EA). Experiments show that formaldehyde diffusion in PMMA is 5.54 × 10^–10^ cm^2^/s at 21 °C (294 K) and increases to 1.27 × 10^–8^ cm^2^/s at 60 °C (333 K).^76^ Because of the lack of experimental data in acrylic paints, we cannot compare these values directly to our simulations since our copolymer binder consists of 40% PMMA + 60% P(EA) or P(*n*BA). However, since PMMA has a much higher glass transition temperature compared to PEA or P(*n*BA) both in experiments and our simulations, this experimental data with slower diffusion of formaldehyde in PMMA with respect to our simulation results in acrylic binder is in good agreement with our expectations.

**Figure 8:**
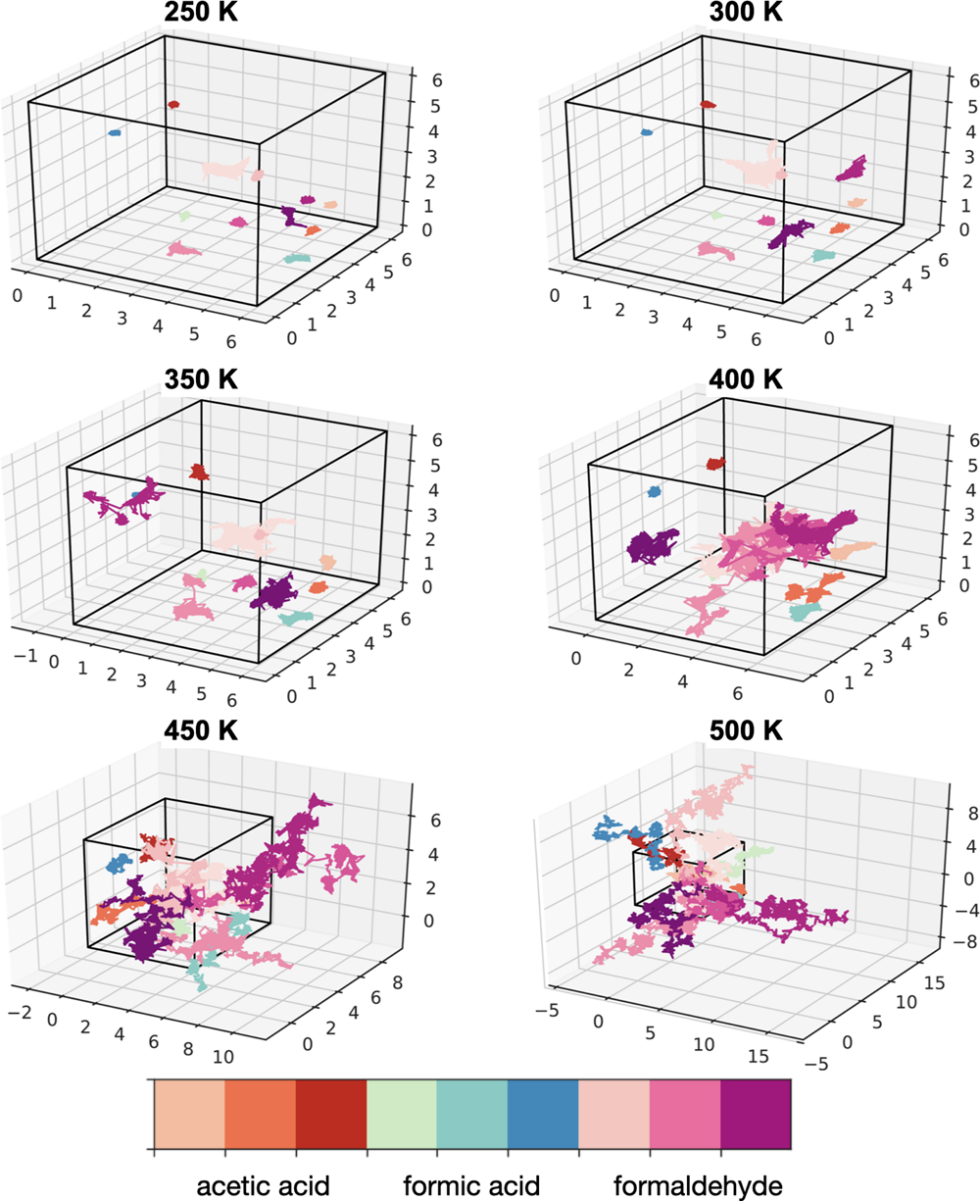
Center-of-mass trajectories of acetic acid, formic acid, and formaldehyde at different temperatures in P(MMA-*co*-EA). See Figure S13 for P(MMA-*co*-*n*BA) results. The equilibrated simulation box size is shown with black lines. All distances (*x*, *y*, *z*) are in nm.

The water self-diffusion coefficient in the acrylic copolymers is similar to that in formaldehyde, which is the most comparable of the three VOCs to water in terms of size.^77^ Whitmore and co-workers measured water diffusion coefficients in different acrylic paints at room temperature and found that they are in the range (1–8) × 10^–7^ cm^2^/s.^78^ We find that the water diffusion is 1.53 × 10^–8^ cm^2^/s in P(MMA-*co*-EA) and 6.88 × 10^–8^ cm^2^/s in P(MMA-*co*-*n*BA) at 300 K. The diffusion of VOCs slightly varies with concentration, but this difference is insignificant. For all VOCs tested, diffusion is slower in P(MMA-*co*-EA), which is consistent with the more favorable solvation energies in P(MMA-*co*-EA) from Table 1. This suggests that the VOCs interact more strongly with P(MMA-*co*-EA) compared with P(MMA-*co*-*n*BA), which results in a slower diffusion in the polymer matrix. Additionally, P(MMA-*co*-*n*BA) has higher solvent accessible surface area (Figure S7), which means there are more empty spaces between the polymer chains that allow for faster diffusion of VOCs. In fact, these empty spaces, termed cages, that result from packing of the polymer chains affect the motion of molecules in a specific way that is often called the cage-breaking mechanism.^79−81^ Here, the particles are trapped and diffuse through small oscillating motions in local cages for a long period of time before they jump into a new cage, which can be seen in our center-of-mass trajectory plots for the VOCs (Figure 8 and Figure S13). The cage-breaking mechanism of pollutant diffusion is also characterized with the intermediate scattering functions in Figure S14, where we observe a two-step relaxation. At low temperatures, slow relaxation over 10 ns means the diffusion of the VOCs is limited to the rattling motion of molecules trapped in cages (β-relaxation). At higher temperatures, the intermediate scattering functions show a faster relaxation (α-relaxation) as the VOC molecules start escaping from the cages. Because of its negative free energy of solvation, water can also be absorbed from the environment into the acrylics through voids in the structure like other VOCs. Once it is absorbed, the diffusion of water resembles that of VOCs and is also hindered by the local cages in the polymer despite the fact that water molecules are less bulky and diffuse faster (Figures S15 and S16).

Because of the cage structure of the polymer, the effect of temperature on diffusion behavior is different in the glassy and rubbery states. Figure 7C,D shows pollutant diffusion plotted against the polymer diffusion at each temperature. In the rubbery state (*T* > *T*_g_), the effect of temperature on VOC and water diffusion is comparable to the effect on polymer diffusion. We observe a linear relationship (with slope close to 1) between pollutant and polymer diffusivities. On the other hand, below the glass transition, diffusion of the VOCs and water is dominated by the local structural barriers imposed by the caging process of the polymers.^82^ The diffusion of the pollutants becomes coupled to the dynamics of the polymer matrix. A VOC or water molecule remains inside a polymer cage until the shape of the cavity changes significantly due to thermal fluctuations, which allow the molecule to hop to another space. This is similar to previously observed strong coupling of phenol diffusion to polymer dynamics in the bisphenol A–polycarbonate melt.^81^

### Intermolecular Interactions

**Figure 9:**
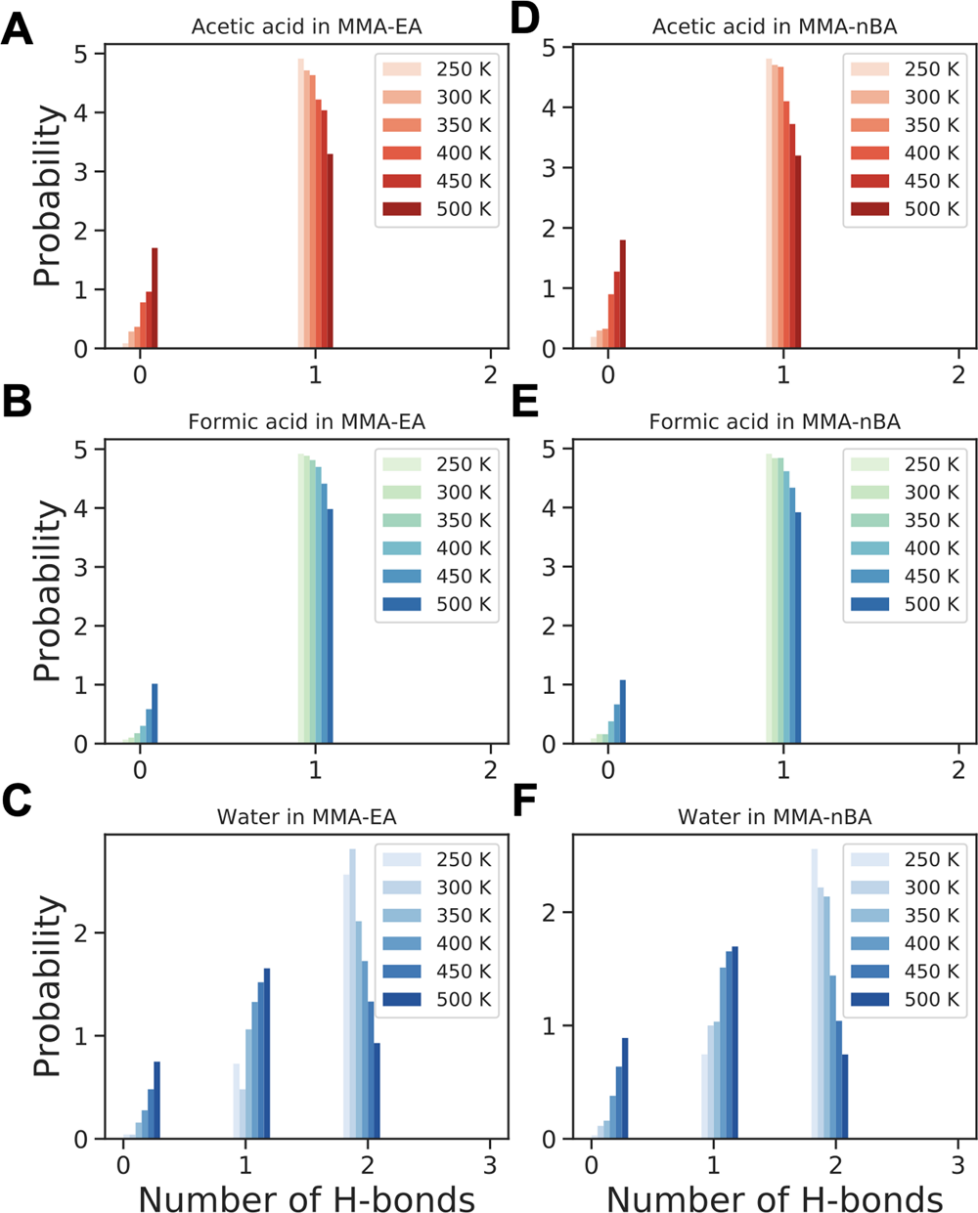
Probability distribution of hydrogen bonding of (A, D) acetic acid, (B, E) formic acid, and (C, F) water with polymer side chains in (A–C) P(MMA-*co*-EA) and (D–F) P(MMA-*co*-*n*BA).

The diffusion mechanism is a consequence of intermolecular interactions of VOCs with polymer chains and other VOCs. The pair distribution functions, *g*(*r*), shown in Figures S17–S23, help us understand the interaction between different molecules. In the *g*(*r*) between VOCs and polymers (Figure S17), which quantify the likelihood of finding a VOC molecule within a certain distance, *r*, from copolymer chains, we observe a small peak at 0.17 nm for acetic acid and formic acid that is due to close interaction of acetic acid and formic acid in the form of hydrogen bonding with the copolymer side chains. Formaldehyde, on the other hand, does not have the OH donor group and lacks the ability to form hydrogen bonds with the polymer side chains, allowing it to diffuse in the copolymer matrix more easily. This idea is also supported by the number of hydrogen bonds between VOCs and polymers (Figure 9), where acetic acid and formic acid make one hydrogen bond with the polymer chains, and an increase in the temperature causes a decrease in the probability of forming hydrogen bonds as a result of increased diffusion restricting the possibility of close contacts that enable hydrogen bonding. The VOCs prefer the monomer with the longer side chain (EA or *n*BA) over MMA when making hydrogen bonds (Figure S24). This observation can be rationalized by the differences in the exposed surface area of each component in the copolymer, shown in Figure S7. While the short side chains of the MMA result in a more compact structure, the longer side chains of PEA and P(*n*BA) give rise to larger spaces between the chains that allow for enhanced VOC interaction. Furthermore, formic acid makes slightly more hydrogen bonds compared to acetic acid (Figure S25). At high concentrations, the VOC interaction with the polymer chains shows the same trends (Figure S26).

Although diffusion of water is similar to formaldehyde in the copolymers due to similarities in the size of water and formaldehyde molecules, water can make two hydrogen bonds due to presence of two donor groups (−OH) that allow it to interact more strongly with the side chains of the polymers. There are essentially two important factors that play a crucial role in determining the diffusion of pollutants in these materials: the ability of pollutants to hydrogen bond to the polymer side chains and the size of the pollutants. While hydrogen bonding with the polymer side chains creates a favorable interaction between the pollutants and polymers and hinders diffusion, the size of the pollutants determines whether these pollutants can easily be absorbed and diffuse through the small spaces between the polymer chains. Hence, while water should be able to diffuse between the local cages that form due to closely packed structure of the copolymers more easily than any of the other VOCs, its favorable interaction via hydrogen bonding limits its diffusion.

The *g*(*r*) between VOCs (Figures S18–S23) suggests that the VOCs only interact with each other at higher temperatures and start to form closer contacts with increased concentration. However, we only observe a very rare occurrence of hydrogen bonding between VOCs at 3000 and 6000 ppm concentrations, where VOCs have a greater chance to come into contact with each other (Figures S27–S29). Water–water interaction is stronger compared to VOC–VOC interactions, where *g*(*r*) shows a peak at small distances as a result of water–water hydrogen bonding (Figure S30). Water–water interactions are stronger in P(MMA-*co*-EA), and we observe more hydrogen bonding compared to P(MMA-*co*-*n*BA) (Figure S31).

### Structural Changes Induced by VOCs and Water

While we do not expect to see a sign of degradation in the acrylics, we studied at these very short time scales; perhaps a more important thing to note is that we can use local changes in the structure as a way to describe the changes that occur in macroscopic properties. One of these properties that may be affected by structural changes induced by presence of VOCs is the glass transition temperature. However, in our simulations with low concentrations of VOCs or water, *T*_g_ does not vary significantly (Figure S33). While we observe a small shift in P(MMA-*co*-EA), there is no clear trend with regard to VOC concentration. This may be a shortcoming of the method we use for calculating *T*_g_, where we take the average total volume of the simulation box that consists of polymers and any pollutants (VOCs, water). Any local structural change brought about from VOC–polymer interaction is not reflected in the volume of our simulation box. Therefore, we are not able to take into account small local changes in structure throughout the simulation box. If we look more closely at the average *R*_e_ and *R*_g_ in Figure S33, we see that the conformations of the polymer chains are different. Although the effect of concentration is not consistent between P(MMA-*co*-EA) and P(MMA-*co*-*n*BA), in both cases any addition of VOCs or water causes the polymers to be less extended and restrict the motion of the chains to adopt more collapsed conformations by forming strong hydrogen bonds with the polymer side chains. Moreover, there are also more empty spaces between the polymer chains (Figure S34) where the VOCs and water diffuse between the chains. This increase in the free volume between the polymer chains might also cause instabilities in the material over long periods of time as a result of local density fluctuations.

**Figure 10:**
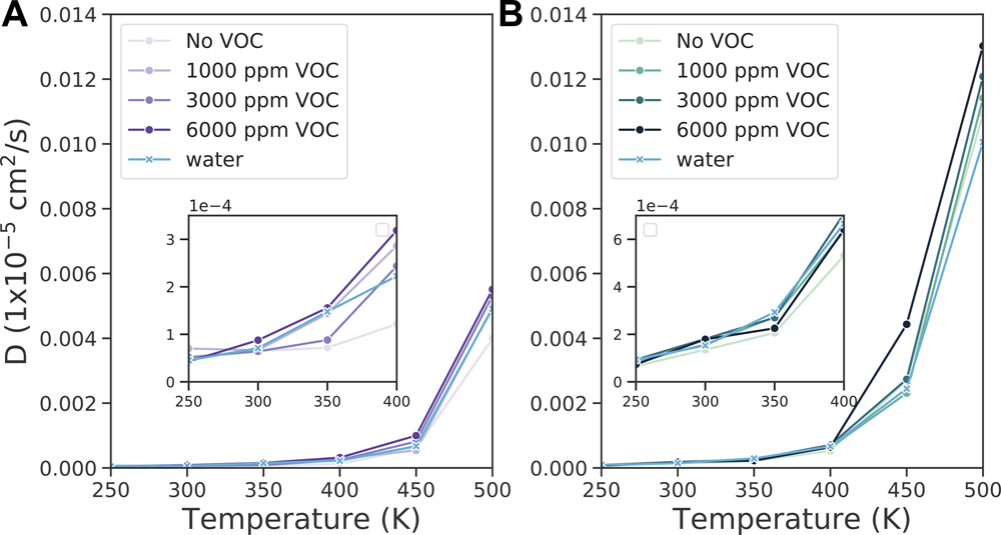
Self-diffusion coefficients of copolymer chains with or without pollutants at different temperatures for (A) P(MMA-*co*-EA) and (B) P(MMA-*co*-*n*BA). The insets correspond to the temperature range of 250–400 K for clarity. Figure S32 shows log(*D*) plotted vs temperature.

Other than static properties, the presence of VOCs or water can also affect the dynamics of the polymer chains. In experiments demonstrating the plasticizing effect of water in polymers, Soleimani et al. found that the increase in water content as a result of high relative humidity caused an increase in the apparent diffusion coefficients of the copolymer P(MMA-*co*-*n*BA).^83^ In our simulations, although the concentration of VOCs and water are low, we already can detect tracers of such effects on the diffusivity of the copolymer chains as shown in Figure 10. Below glass transition, the effect for our system parameters is too small to detect a clear trend with respect to VOC concentration. Above *T*_g_ (*T* > 400 K), polymer diffusion is slightly faster with VOCs at higher temperatures.

## Conclusions

The underlying mechanism associated with acrylic paints’ degradation is far from being understood. In this context, computational approaches contribute to filling the gap between fundamental understanding and practical experience. However, different energy and time scales involved in the relevant physical processes, leading to important structural changes, require the use of multiscale computational tools. To this aim, we developed an atomistic model to study the microscopic structure of acrylic binders and their interactions with impurities, such as VOCs and water, to understand the changes that occur at very short time scales on a molecular level. On the basis of the results of our atomistic model, we suggest that intermolecular interactions, such as hydrogen bonding, play a crucial role and must be included when developing nonbonded potentials in the coarse-grained scale. Also, the methyl group in PMMA should be accounted for since it significantly affects the density and packing of the polymer chains whereas other chemical details, such as backbone and side chains, can be omitted. For the development of continuum model, some of the important properties that should be transferred are density, diffusion, and porosity of the polymers. It is also necessary to consider diffusion of pollutants at short and long times.

From atomistic simulations we extract structural (scattering) and thermodynamic (glass transition temperature) information that can be directly compared to experimental measurements. Furthermore, self-diffusion coefficients enable us to compare differences in diffusion of the VOCs and identify the conditions at which they get trapped in the material. Our simulations suggest that small modifications in the chemical moieties of acrylics (i.e., the length of polymer side chains) can result in differences in structure as a result of exposure to variations in the environmental conditions. Additionally, to access longer time and length scales, we will use this information in future to parametrize coarse-grained and continuum models that include a sufficient amount of chemical detail to make predictions about polymer lifetime and degradation and draw a useful comparison with experiments.
