# Supplementary material for: Acrylic Paints: An Atomistic View of Polymer Structure and Effects of Environmental Pollutants
Source: J Phys Chem B. 2021 Sep 15;125(38):10854–65. doi: 10.1021/acs.jpcb.1c05188 (PMC8488938; doi:10.1021/acs.jpcb.1c05188)
Supplement: Supplementary file 1 — jp1c05188_si_001.pdf [file jp1c05188_si_001.pdf]

Acrylic Paints: An Atomistic View of Polymer Structure and Effects of Environmental Pollutants

Aysenur Iscen, Nancy C. Forero-Martinez, Omar Valsson, and Kurt Kremer*

Max Planck Institute for Polymer Research, Ackermannweg 10, 55128 Mainz, Germany

E-mail: kremer@mpip-mainz.mpd.de

Supporting figures for atomistic model and force field validation

In order to evaluate the accuracy of our model, we performed simulations with GAFF and OPLS forcefields. The results obtained with GAFF are reported in the main text. In the following figure, we show the Tg and SAXS using OPLS force field. Diffusion coefficients of polymer chains with OPLS is given in Figure 2 of main text.

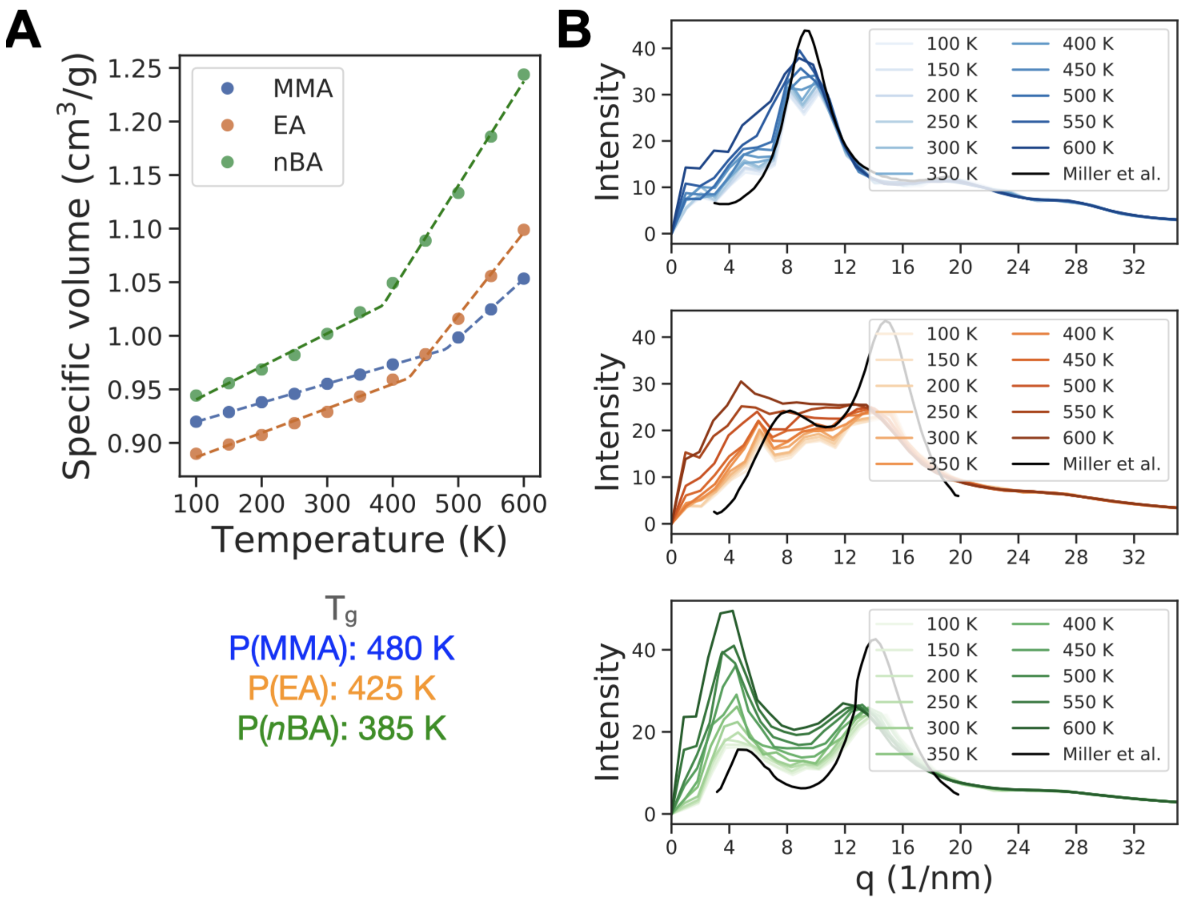

Figure S1: (**A**) Specific volume and glass transition temperatures and (**B**) small angle X-ray scattering structure factor of PMMA, PEA, P(nBA) calculated using OPLS force field.

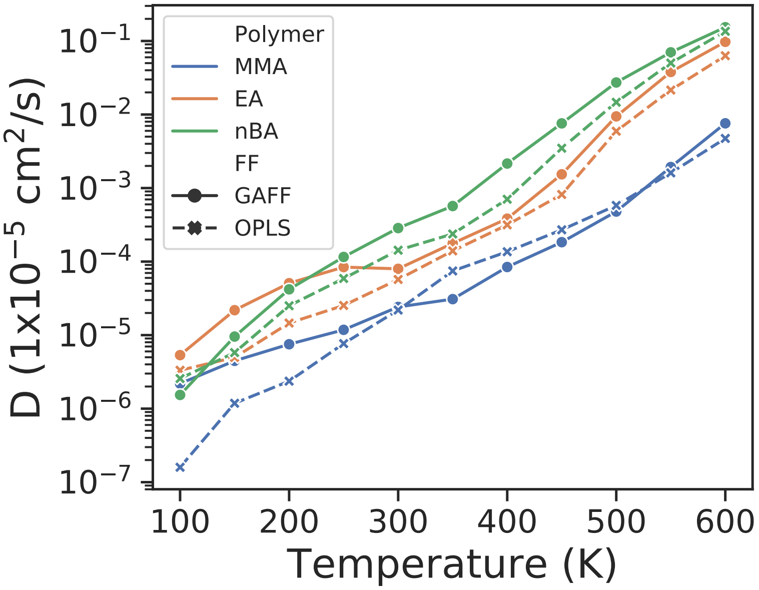

Figure S2: The log of self-diffusion coefficients of PMMA, PEA, P(nBA) calculated using GAFF (solid lines) and OPLS (dashed lines) as a function of temperature.

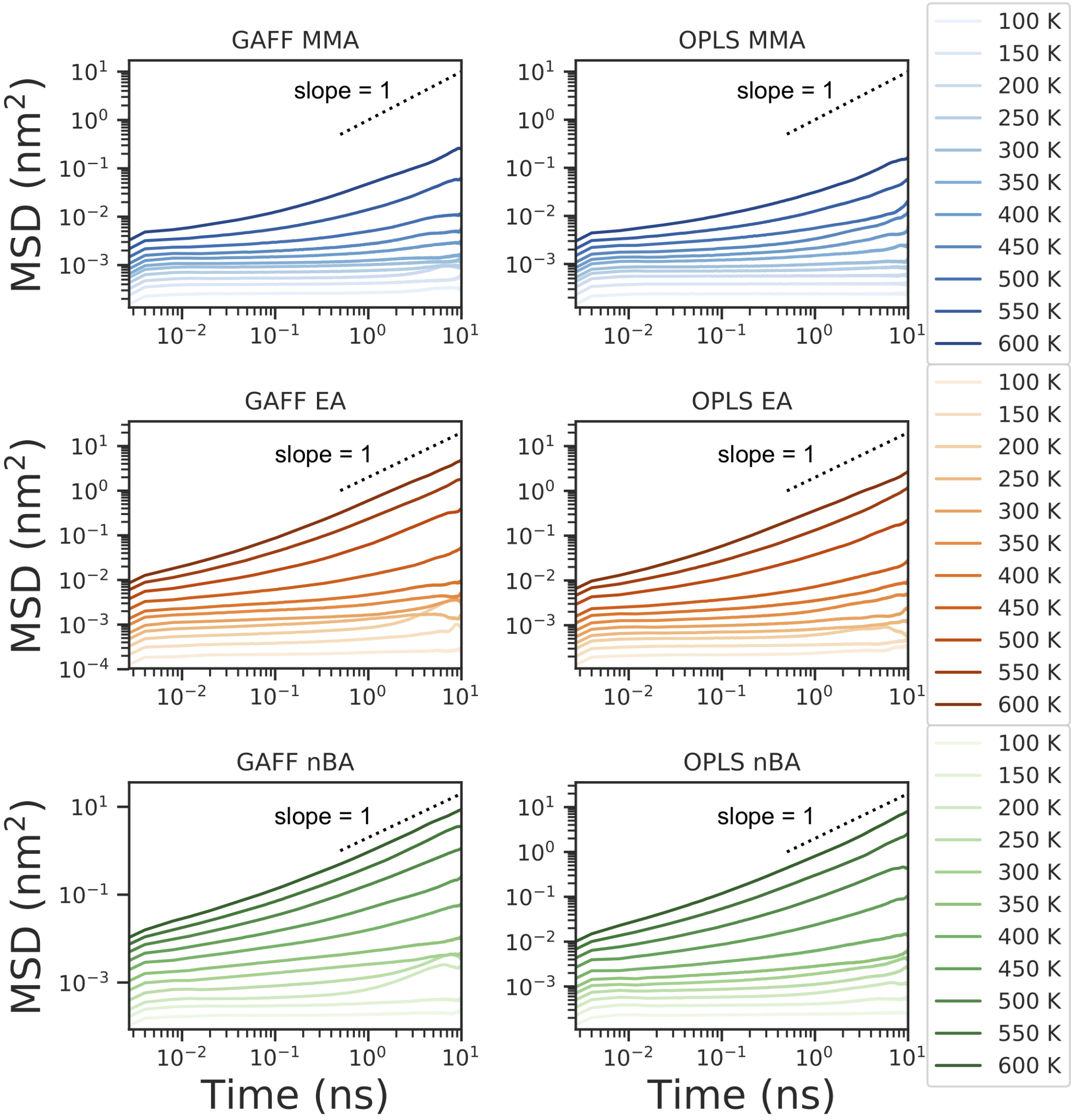

Figure S3: The mean square displacement of center of mass of 15mer PMMA, PEA and P(nBA) over last 10 ns of trajectory. A line with slope of 1 is plotted to guide the eye.

Supporting figures for properties of acrylics found in paints

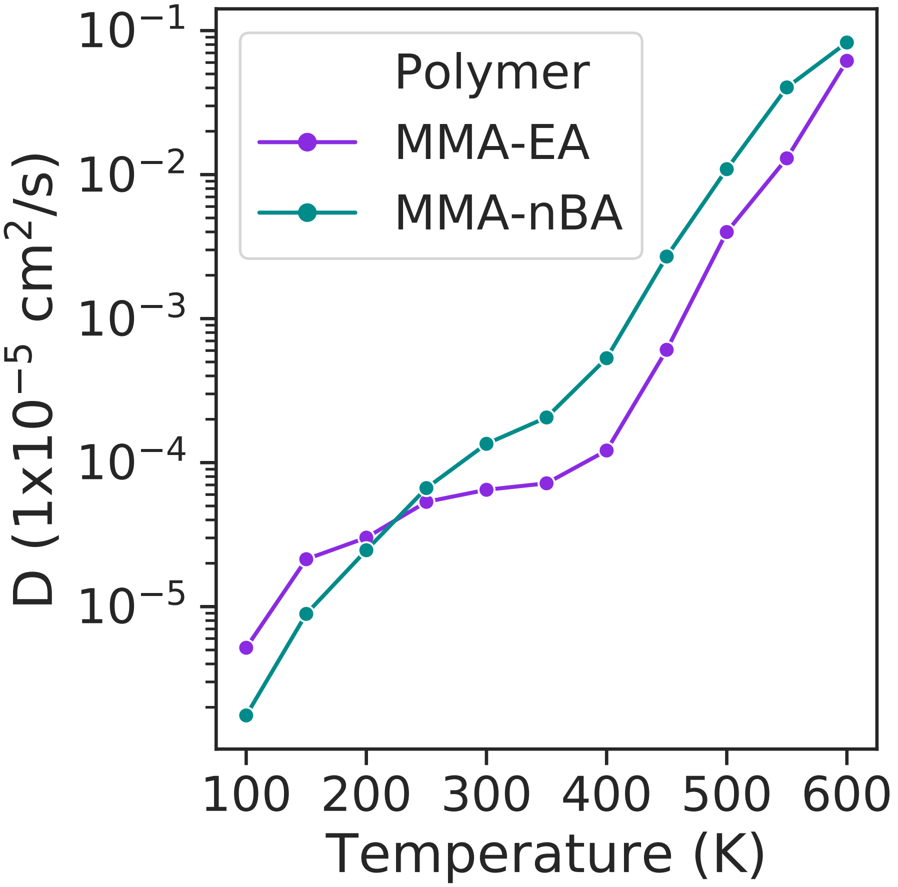

Figure S4: The log of self-diffusion coefficients of P(MMA-co-EA) and P(MMA-co-*n*BA) as a function of temperature.

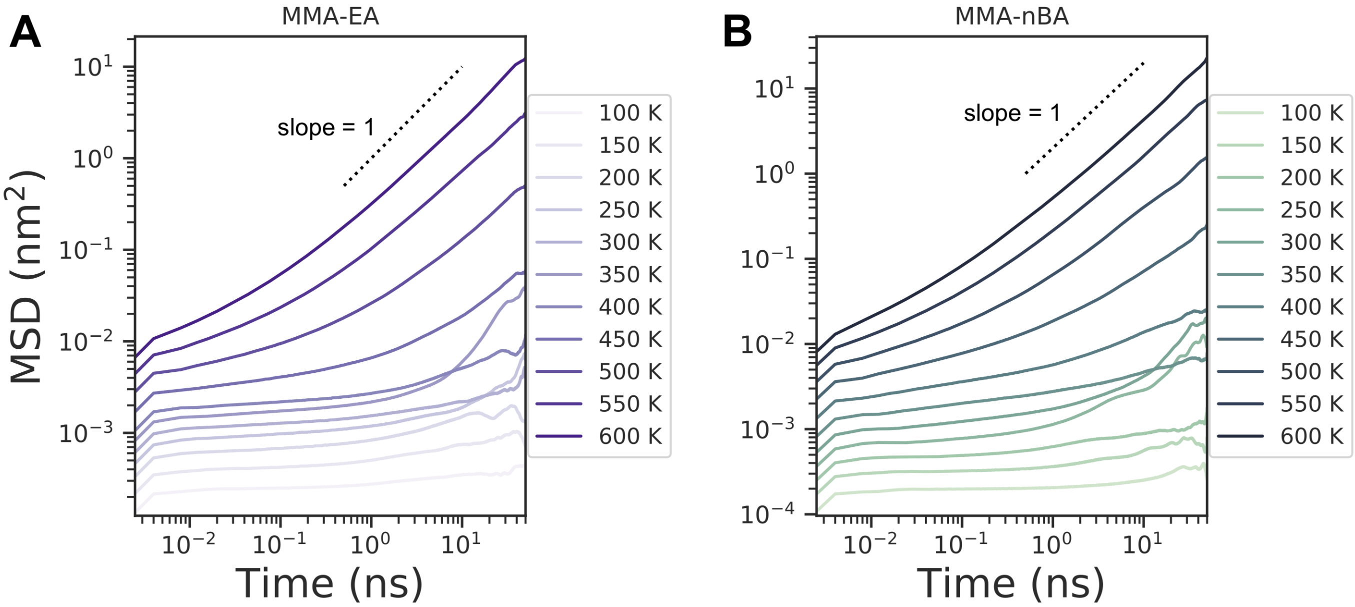

Figure S5: The mean square displacement of center of mass of 15mer **(A)** P(MMA-co-EA) and **(B)** P(MMA-co-nBA) over last 50 ns of trajectory. A line with slope of 1 is plotted to guide the eye.

For each copolymer P(MMA-co-EA) and P(MMA-co-nBA), we show the contribution of different monomers MMA, EA and nBA to the overall structure.

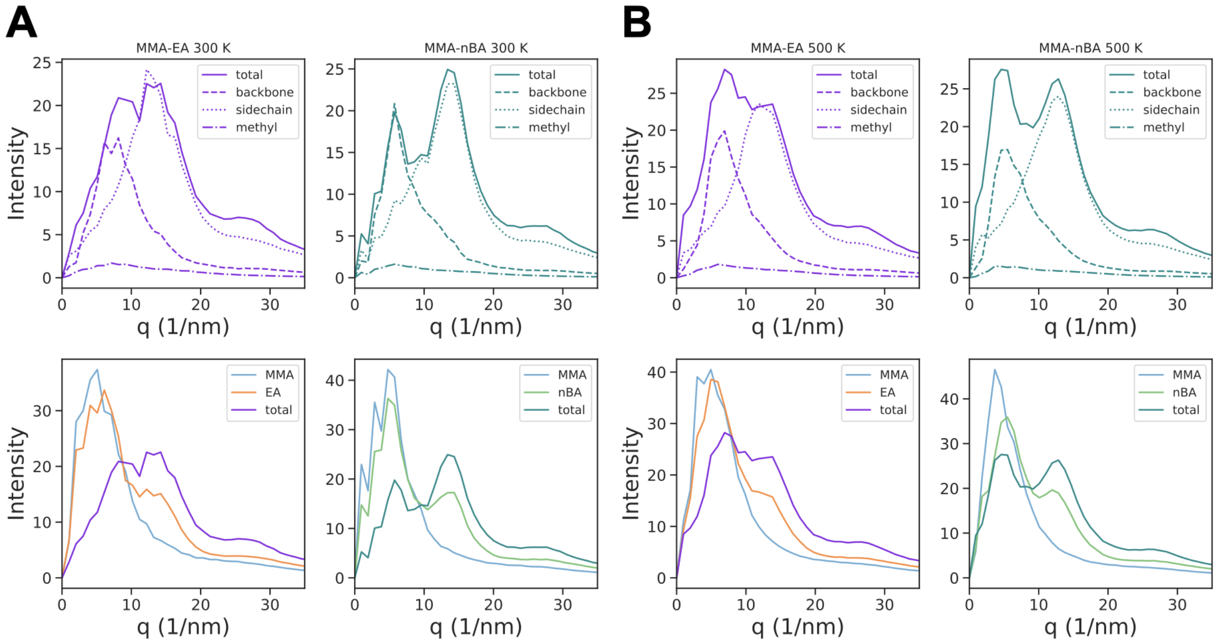

Figure S6: Contributions of polymer backbone, side chain, methyl group and monomers to the small angle x-ray scattering (SAXS) spectra for P(MMA-co-EA) and P(MMA-co-nBA) at 300 K (**A**) and 500 K (**B**).

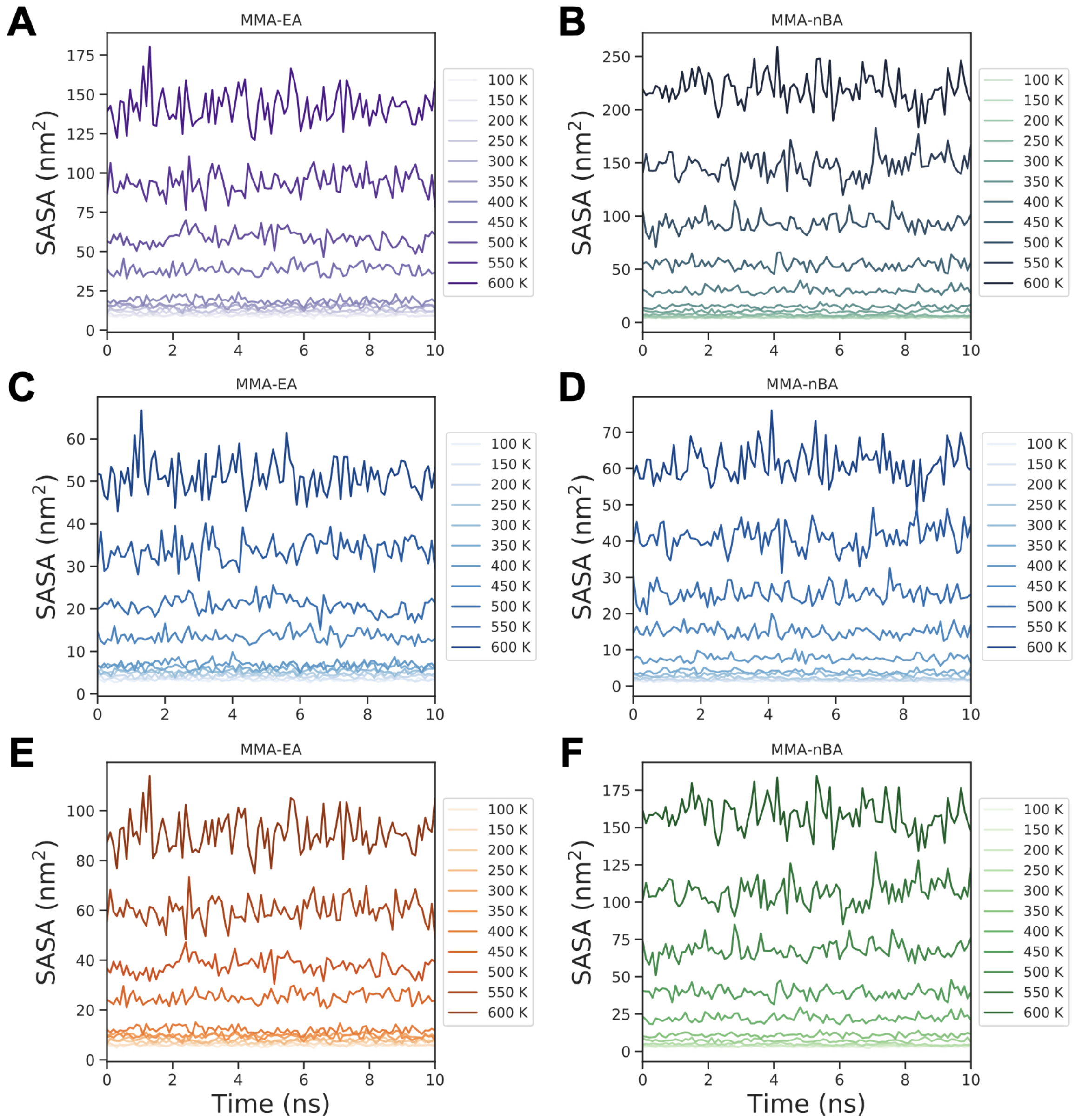

Figure S7: Total Solvent accessible surface area (SASA) calculated for **(A)** P(MMA-co-EA) and **(B)** P(MMA-co-*n*BA) at different temperatures. **(C,D)** SASA for MMA, **(E)** PEA and **(F)** P(*n*BA) component of each copolymer. Only data from the last 10 ns of 100 ns simulation is shown.

Supporting figures for effects of VOCs and water on acrylic structure

Diffusion mechanisms

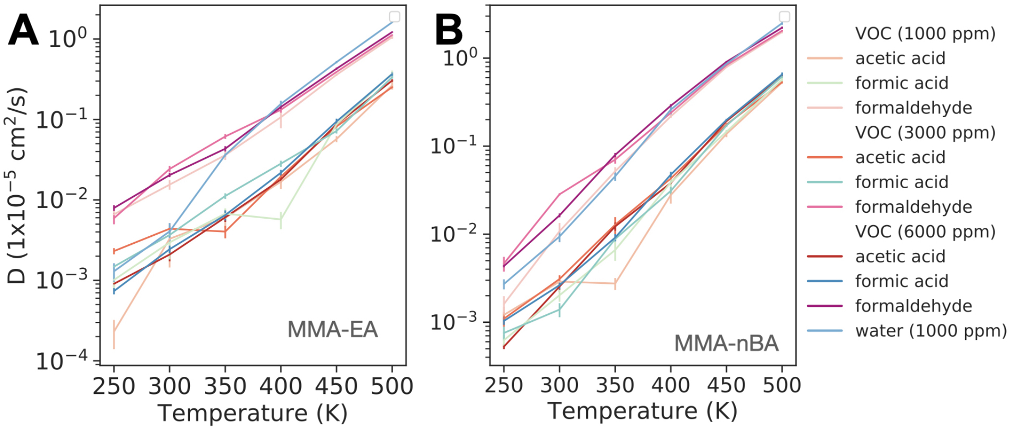

Figure S8: The log of self-diffusion coefficients of acetic acid, formic acid, formaldehyde and water in **(A)** P(MMA-co-EA) and **(B)** P(MMA-co-nBA) as a function of temperature.

Mean square displacement (MSD) of each pollutant molecule (acetic acid, formic acid, formaldehyde, water) is used to calculate the diffusion coefficients, D, as a function of temperature. The MSD shown below are for a single 10 ns trajectory. In our calculation of average D, we divided a 40 ns trajectory into 4 blocks and took the mean of D calculated from 0-10, 10-20, 20-30, 30-40 and 0-40 ns.

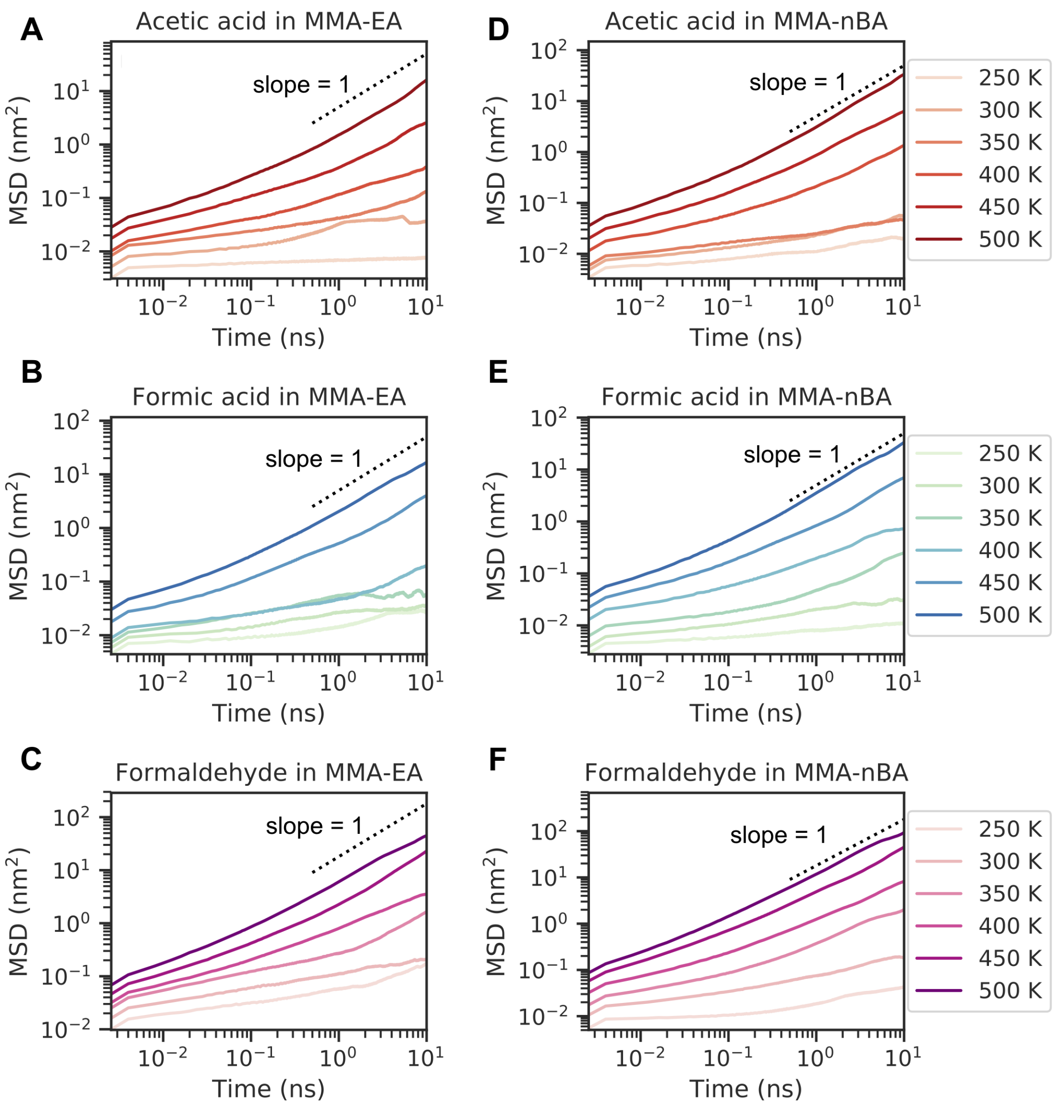

Figure S9: The mean square displacement of center of mass of 1000 ppm acetic acid, formic acid and formaldehyde in **(A-C)** P(MMA-co-EA) and **(D-F)** P(MMA-co-nBA) over 10 ns of trajectory. A line with slope of 1 is plotted to guide the eye.

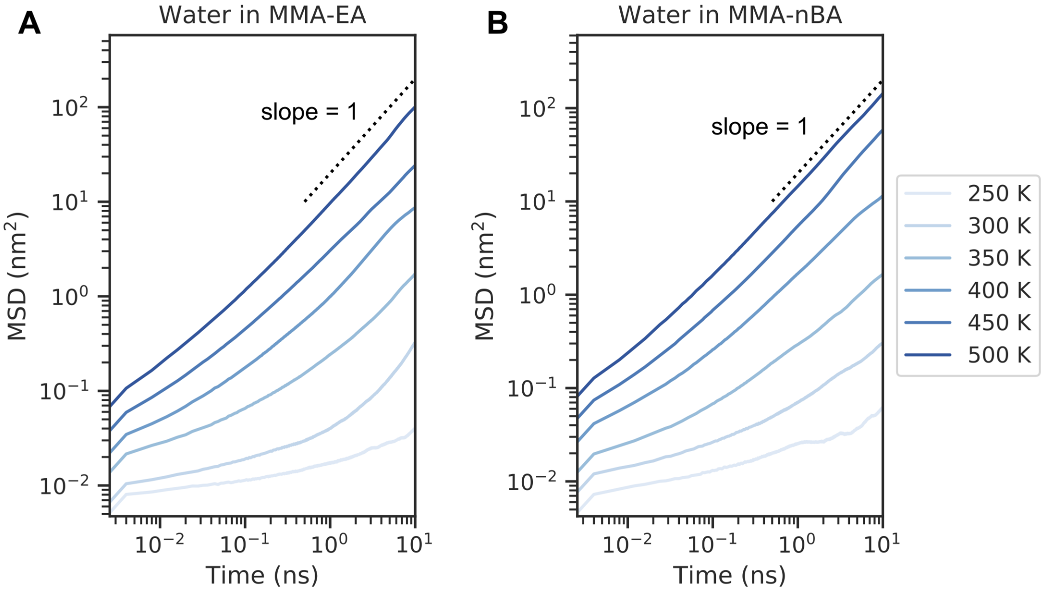

Figure S10: The mean square displacement of center of mass of 1000 ppm water in **(A)** P(MMA-co-EA) and **(B)** P(MMA-co-*n*BA) over 10 ns of trajectory. A line with slope of 1 is plotted to guide the eye.

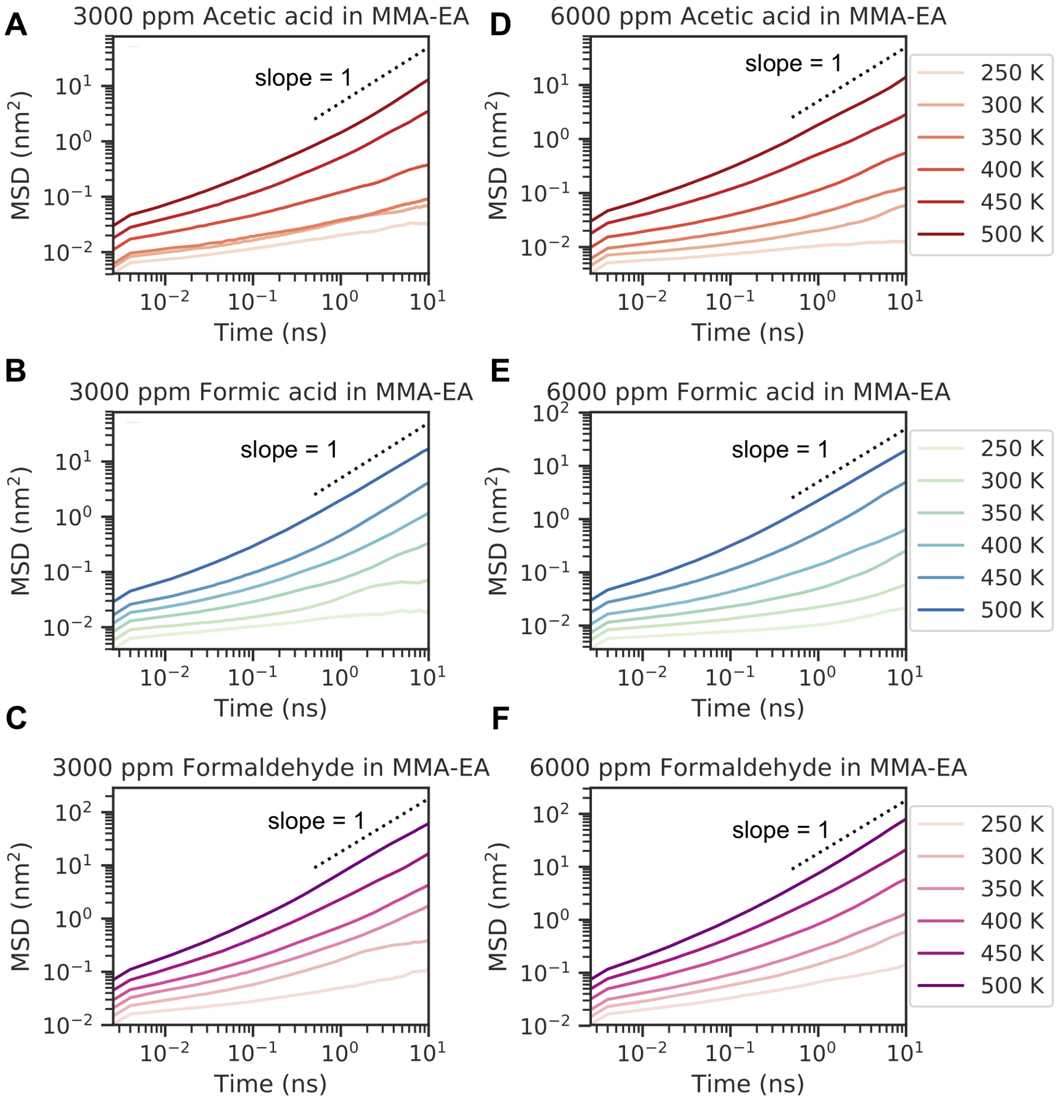

Figure S11: The mean square displacement of center of mass of **(A-C)** 3000 ppm and **(D-F)** 6000 ppm acetic acid, formic acid and formaldehyde in P(MMA-co-EA) over 10 ns of trajectory. A line with slope of 1 is plotted to guide the eye.

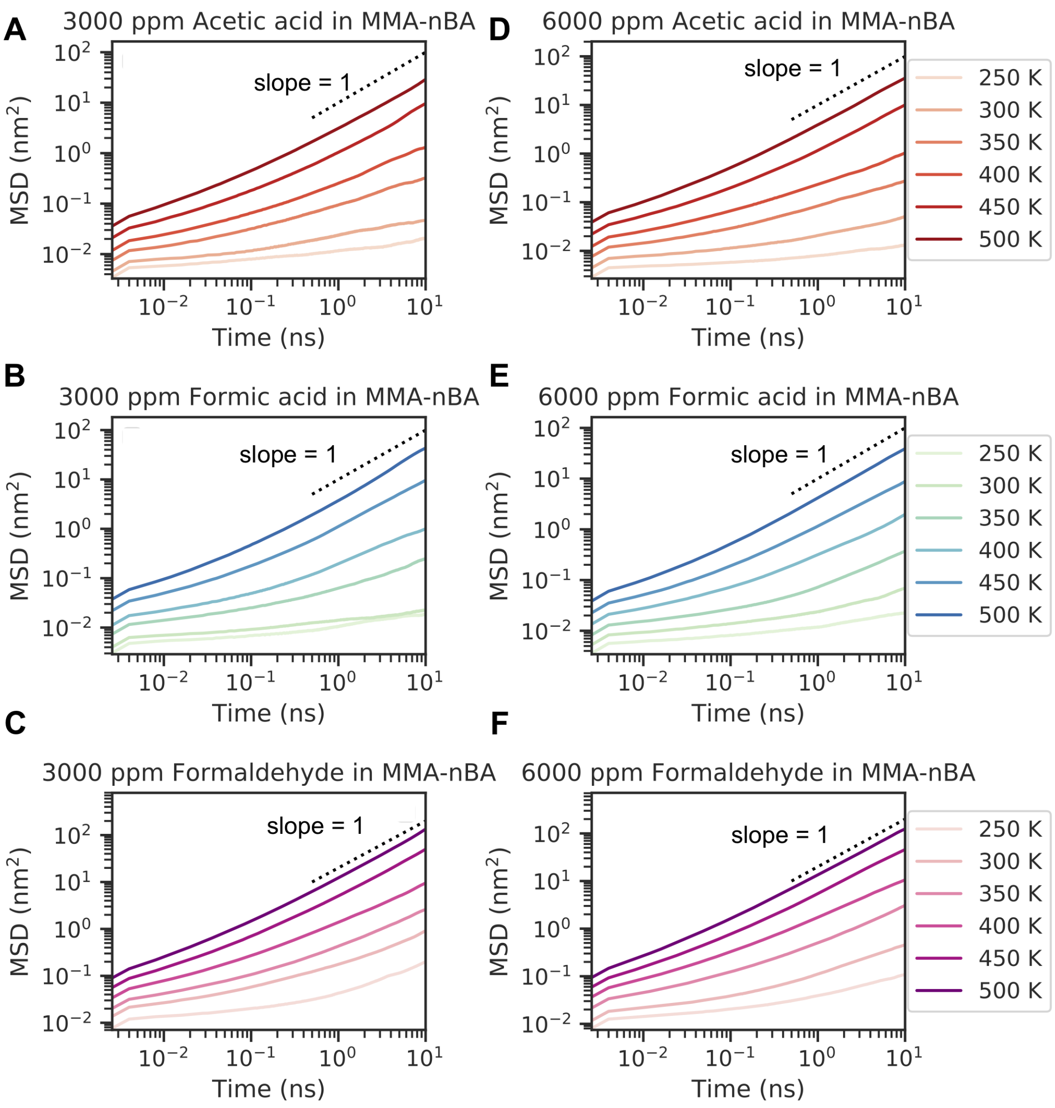

Figure S12: The mean square displacement of center of mass of **(A-C)** 3000 ppm and **(D-F)** 6000 ppm acetic acid, formic acid and formaldehyde in P(MMA-co-*n*BA) over 10 ns of trajectory. A line with slope of 1 is plotted to guide the eye.

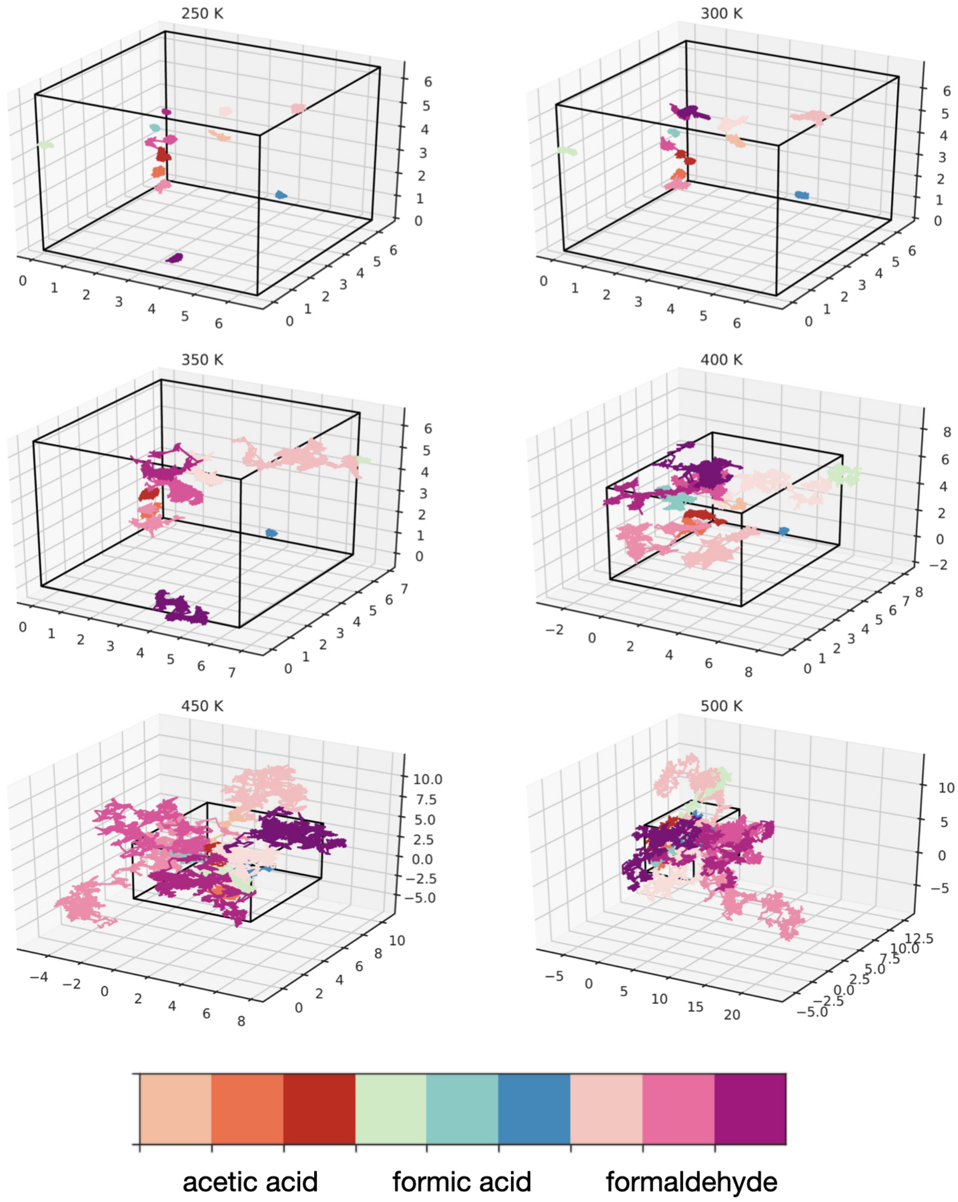

Figure S13: The center of mass trajectories of acetic acid, formic acid and formaldehyde at different temperatures in P(MMA-co-nBA). The equilibrated simulation box size is shown with black lines. All distances (x,y,z) are in nm.

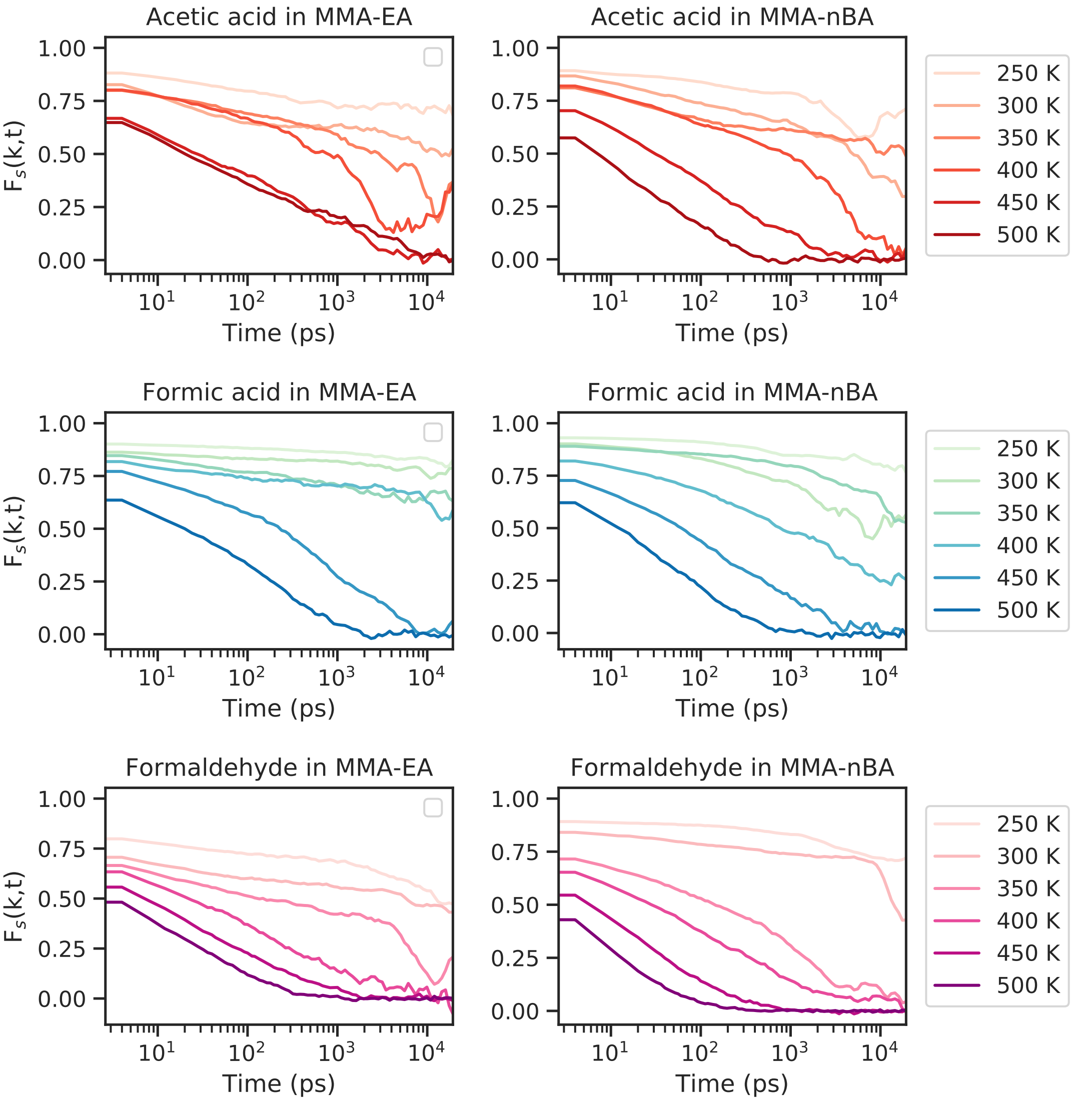

Figure S14: Self part of intermediate scattering functions for acetic acid, formic acid and formaldehyde (1000 pppm concentrations) at different temperatures in P(MMA-co-EA) and P(MMA-co-nBA). The F_s is evaluated at k=q_{max} (first peak) in the structure factors individually for each temperature.

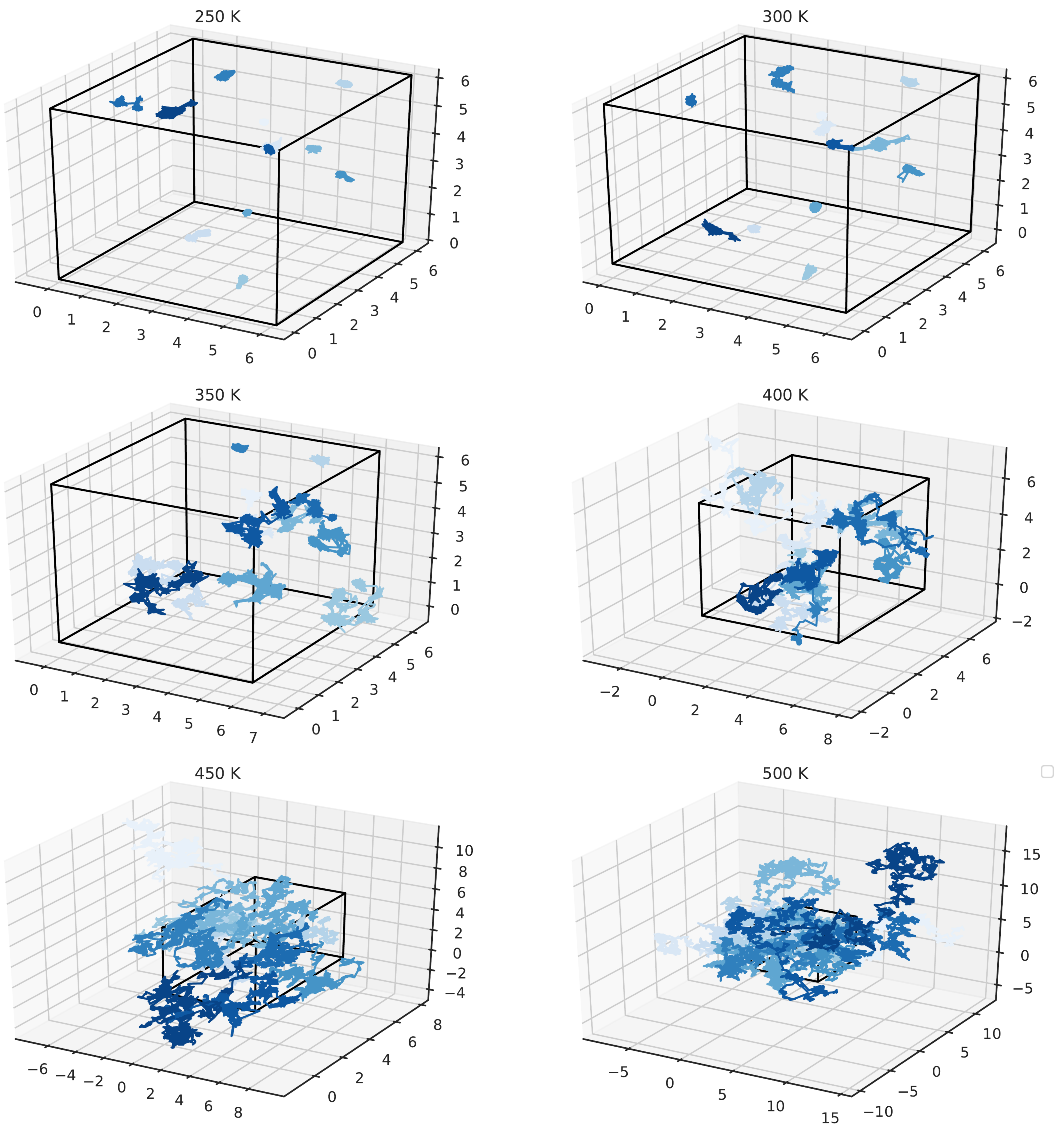

Figure S15: The center of mass trajectories of water at different temperatures in P(MMA-co-EA). The equilibrated simulation box size is shown with black lines. All distances (x,y,z) are in nm.

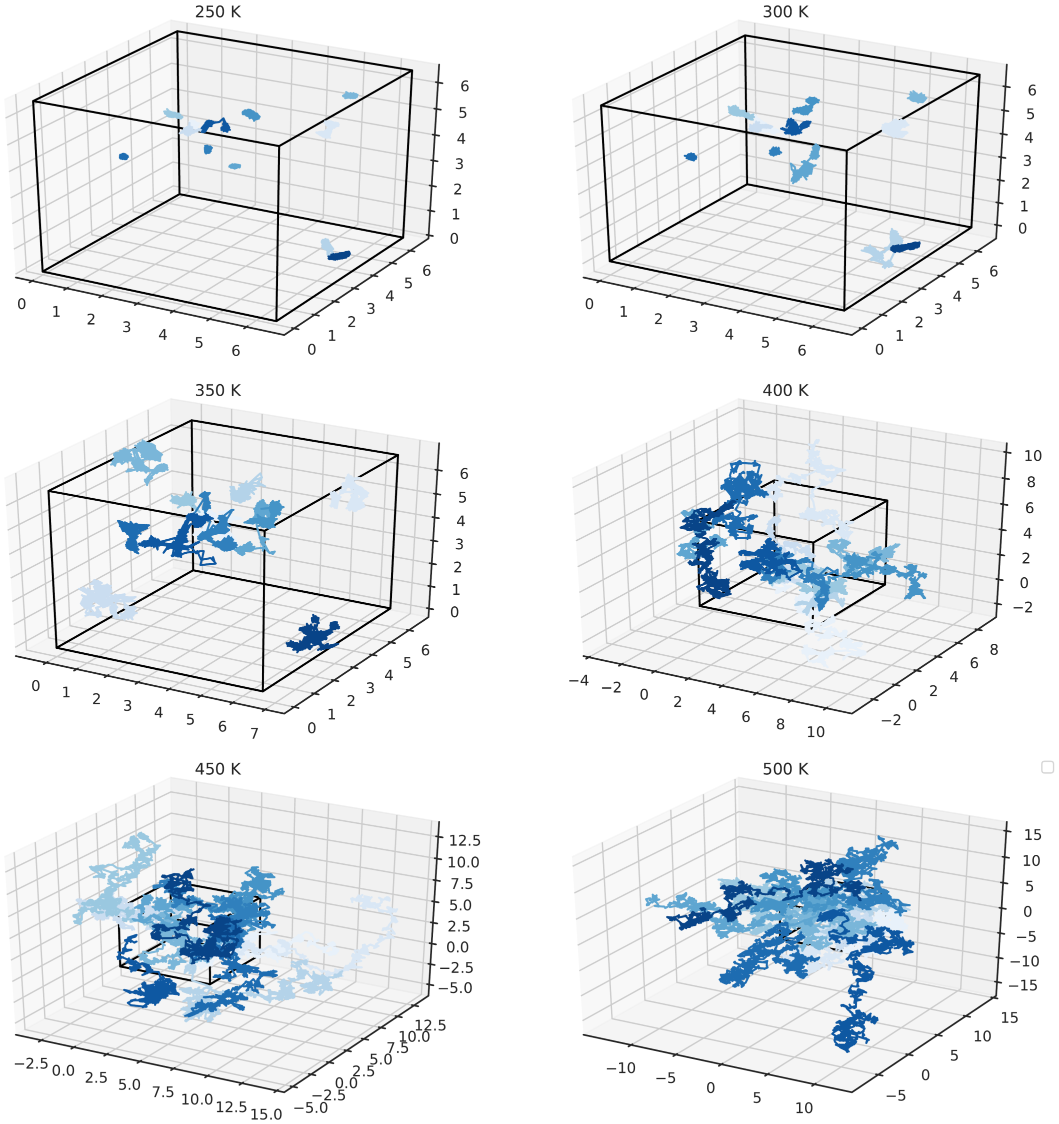

Figure S16: The center of mass trajectories of water at different temperatures in P(MMA-co-nBA). The equilibrated simulation box size is shown with black lines. All distances (x,y,z) are in nm.

Intermolecular interactions

Radial distribution functions

Pair distribution functions for VOC - polymer interaction:

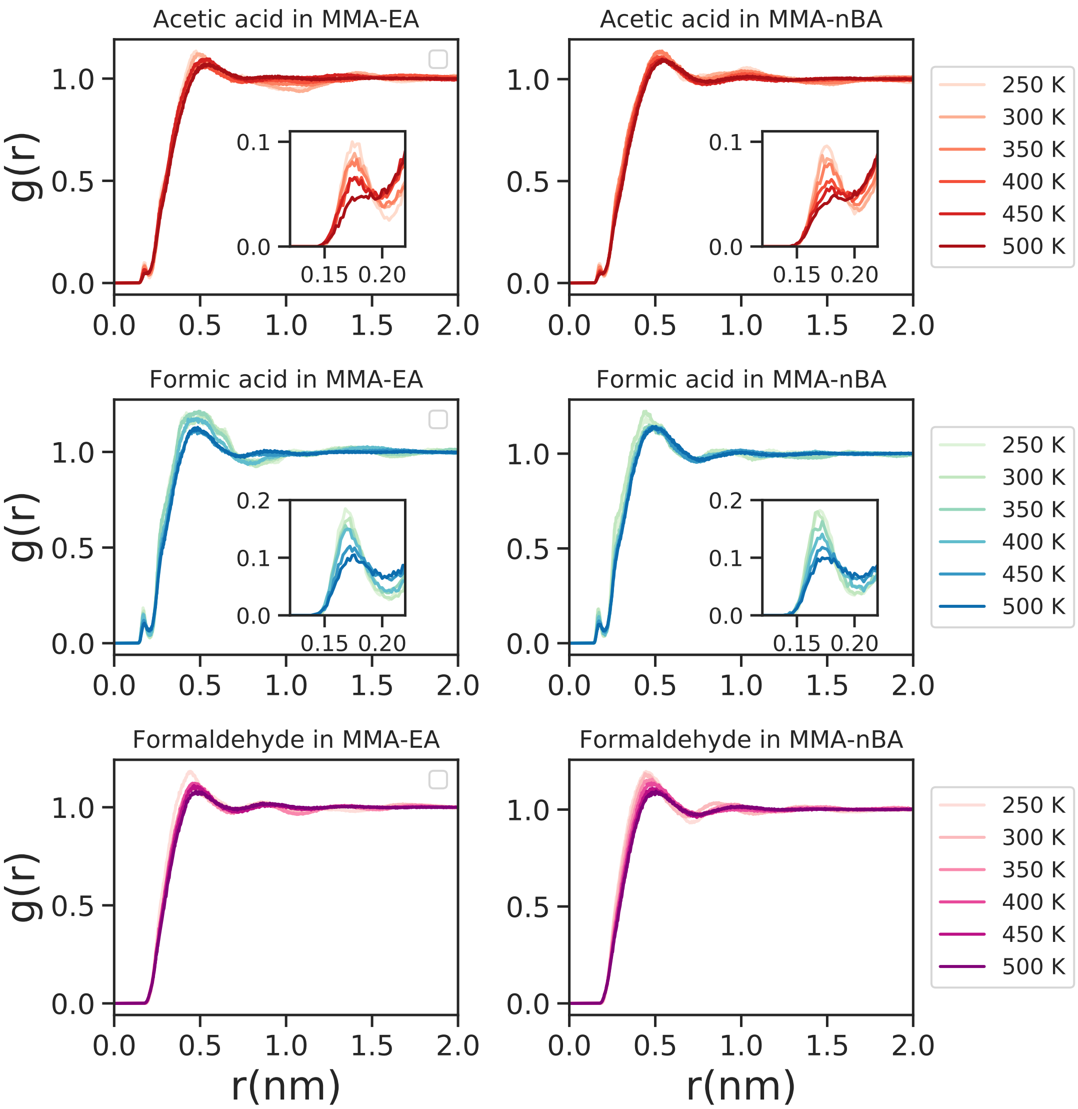

Figure S17: The pair distribution functions, g(r), between VOCs and copolymer chains for acetic acid, formic acid and formaldehyde in P(MMA-co-EA) and P(MMA-co-nBA) at different temperatures.

Pair distribution functions for VOC-VOC interactions:

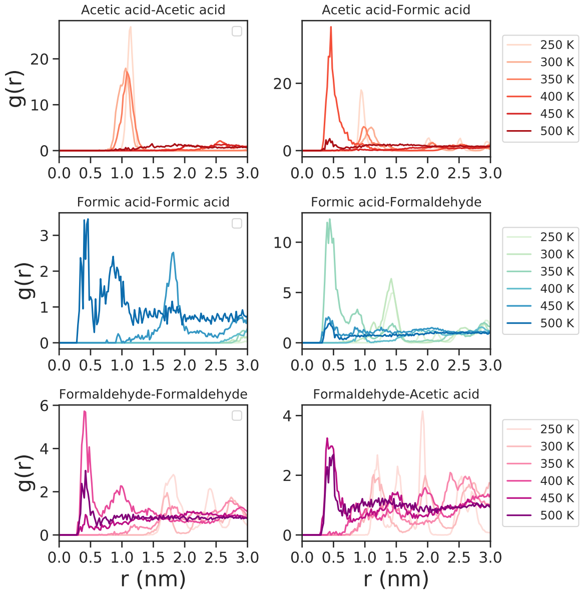

Figure S18: The pair distribution functions, g(r), between VOCs and other VOCs for 1000 ppm acetic acid, formic acid and formaldehyde in P(MMA-co-EA) at different temperatures.

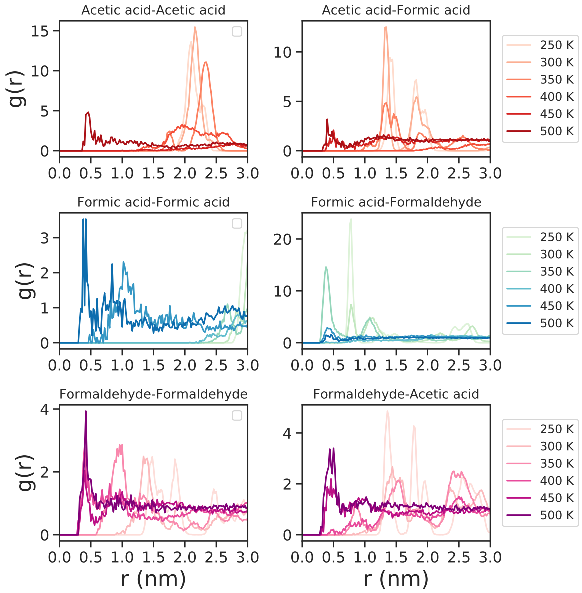

Figure S19: The pair distribution functions, g(r), between VOCs and other VOCs for 1000 ppm acetic acid, formic acid and formaldehyde in P(MMA-co-nBA) at different temperatures.

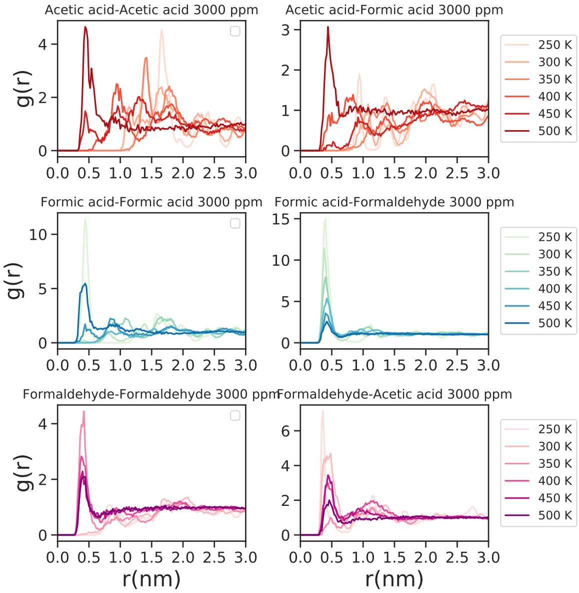

Figure S20: The pair distribution functions, g(r), between VOCs and other VOCs for 3000 ppm acetic acid, formic acid and formaldehyde in P(MMA-co-EA) at different temperatures.

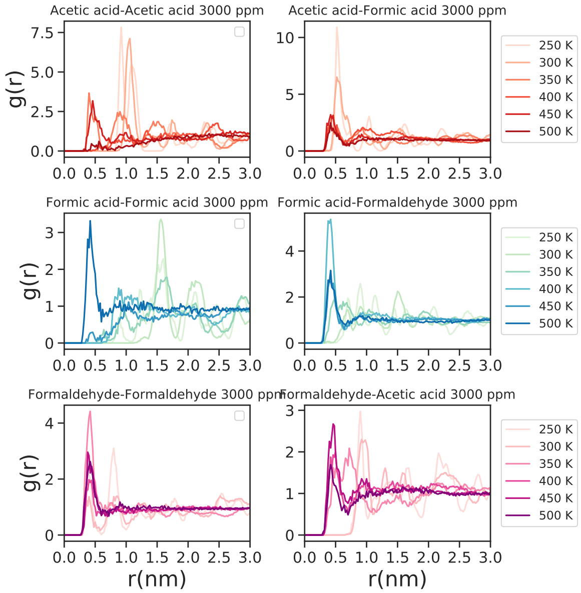

Figure S21: The pair distribution functions, g(r), between VOCs and other VOCs for 3000 ppm acetic acid, formic acid and formaldehyde in P(MMA-co-nBA) at different temperatures.

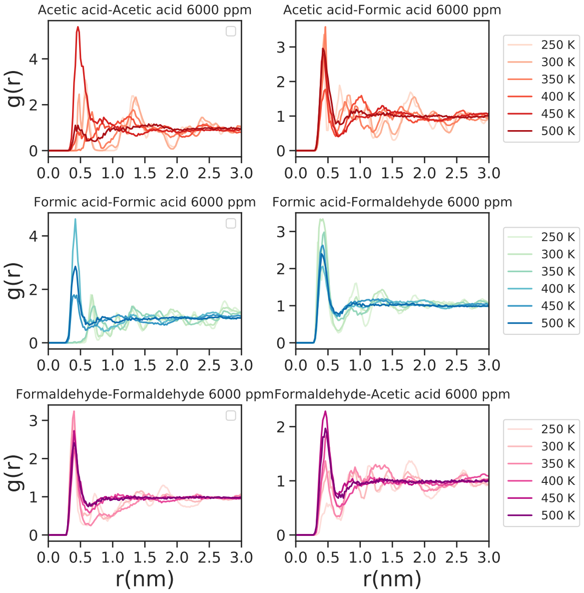

Figure S22: The pair distribution functions, g(r), between VOCs and other VOCs for 6000 ppm acetic acid, formic acid and formaldehyde in P(MMA-co-EA) at different temperatures.

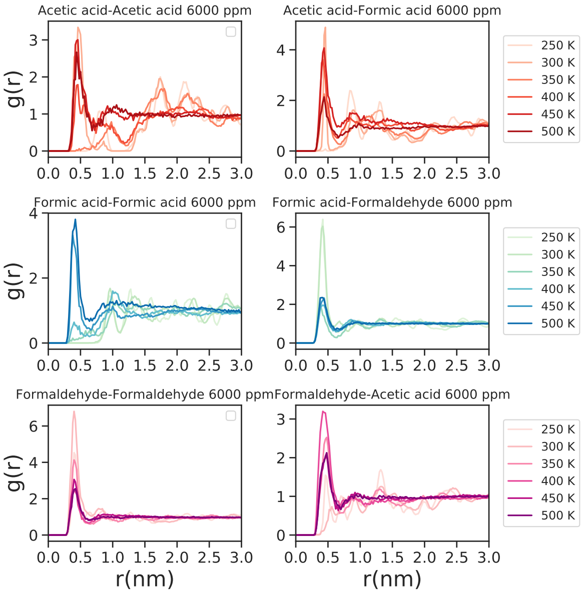

Figure S23: The pair distribution functions, g(r), between VOCs and other VOCs for 6000 ppm acetic acid, formic acid and formaldehyde in P(MMA-co-nBA) at different temperatures.

Hydrogen bonding

VOC - polymer hydrogen bonding interactions:

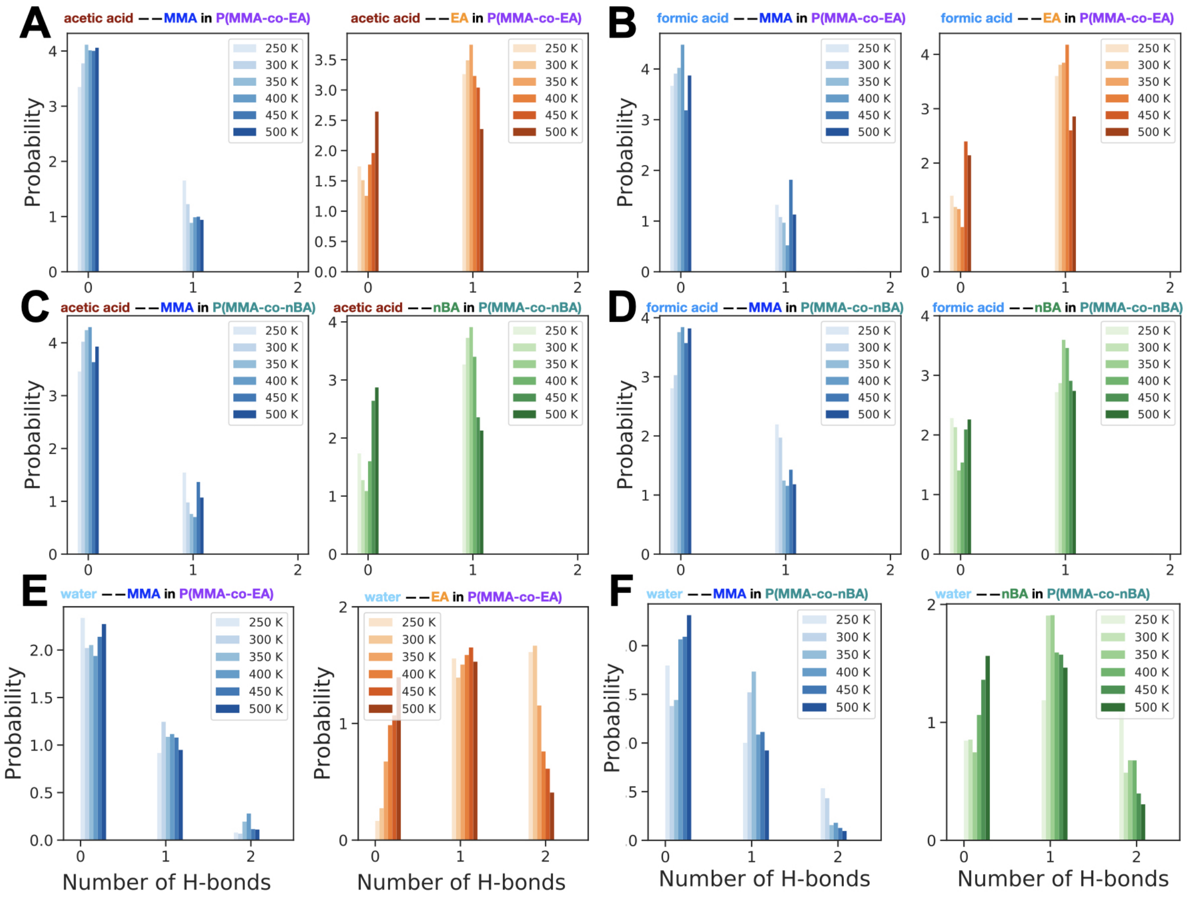

Figure S24: Probability distribution of number of hydrogen bonds between (**A,C**) acetic acid and MMA, EA, *n*BA, (**B,D**) formic acid and MMA, EA, *n*BA and (**E,F**) water and MMA, EA, *n*BA as a function of temperature.

VOC - VOC hydrogen bonding interactions:

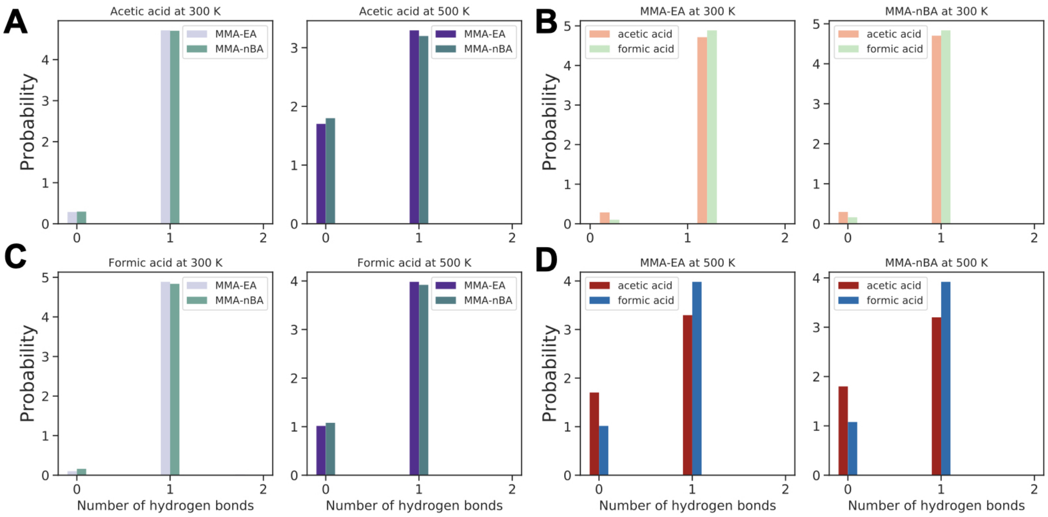

Figure S25: Probability distribution of number of hydrogen bonds between VOCs and copolymer chains, comparing (A) acetic acid and formic acid and (B) P(MMA-co-EA) and P(MMA-co-nBA) at 300 K and 500 K.

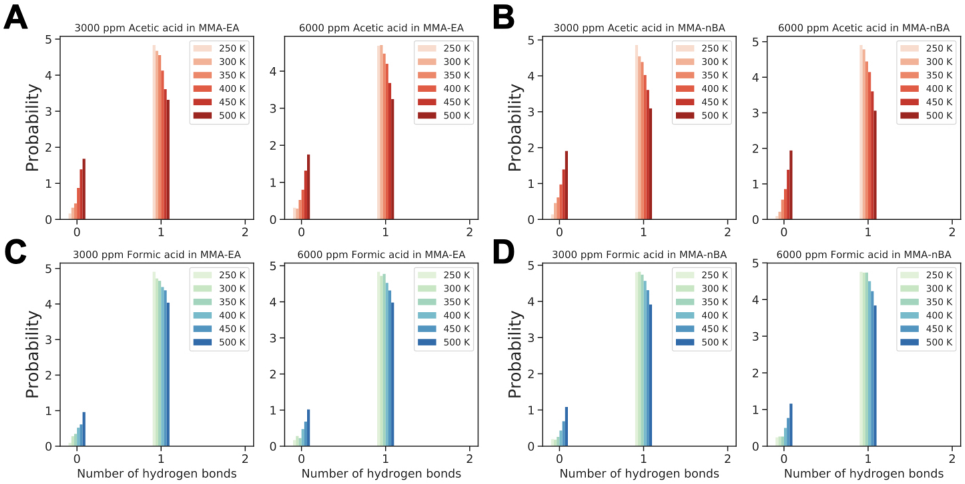

Figure S26: The probability distribution of the number of hydrogen bonds between high concentrations of acetic acid and formic acid and (**A,C**) P(MMA-co-EA) or (**B,D**) P(MMA-co-nBA) at different temperatures.

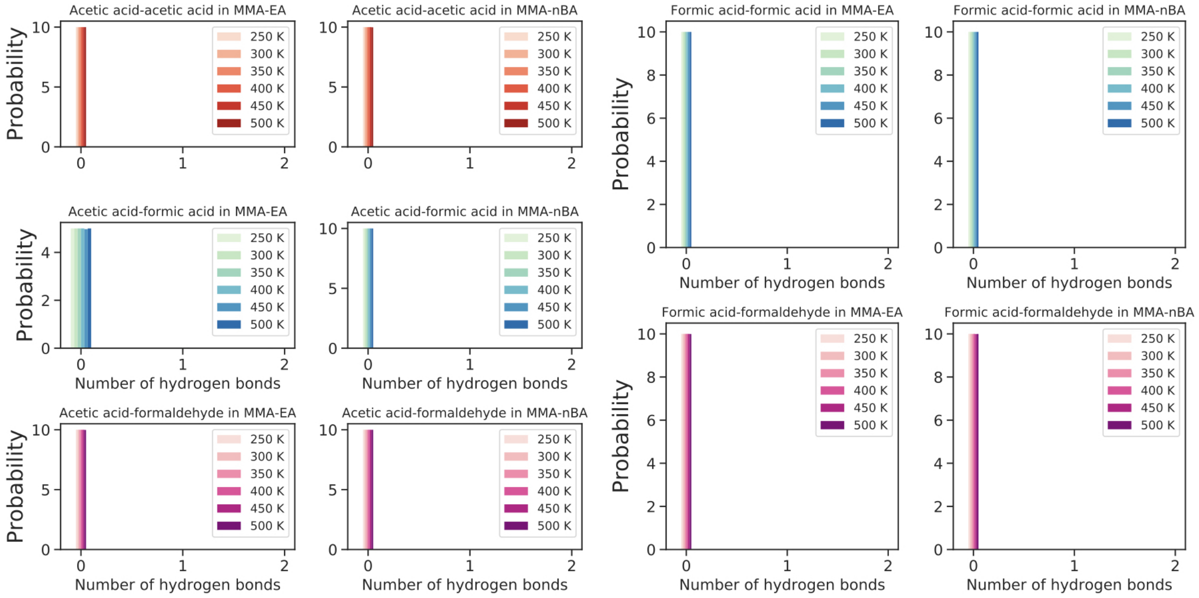

Figure S27: Probability distribution of number of VOC-VOC hydrogen bonds for 1000 ppm concentration.

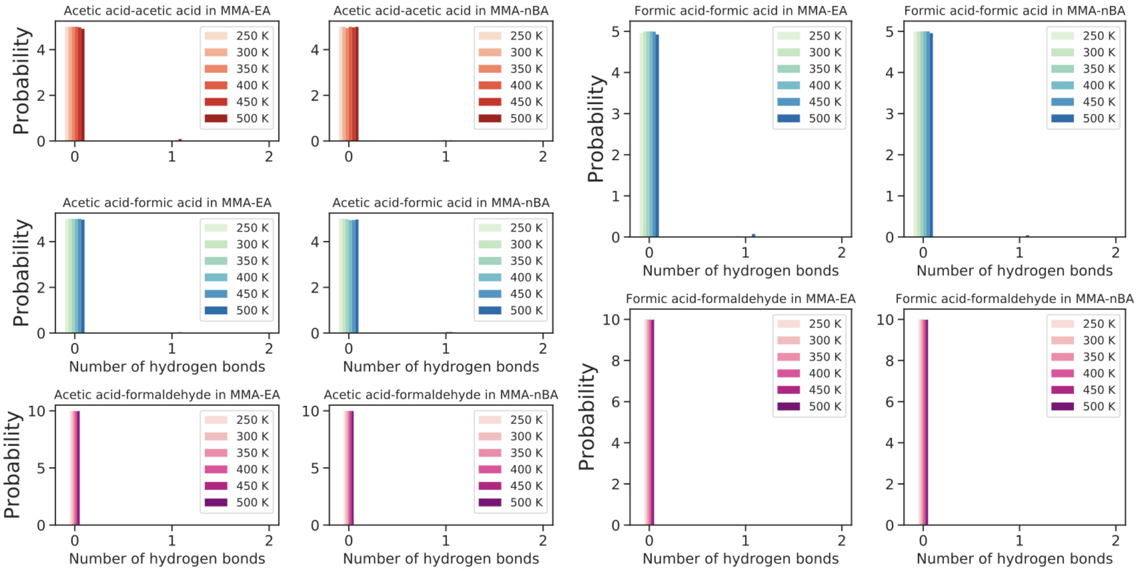

Figure S28: Probability distribution of number of VOC-VOC hydrogen bonds for 3000 ppm concentration.

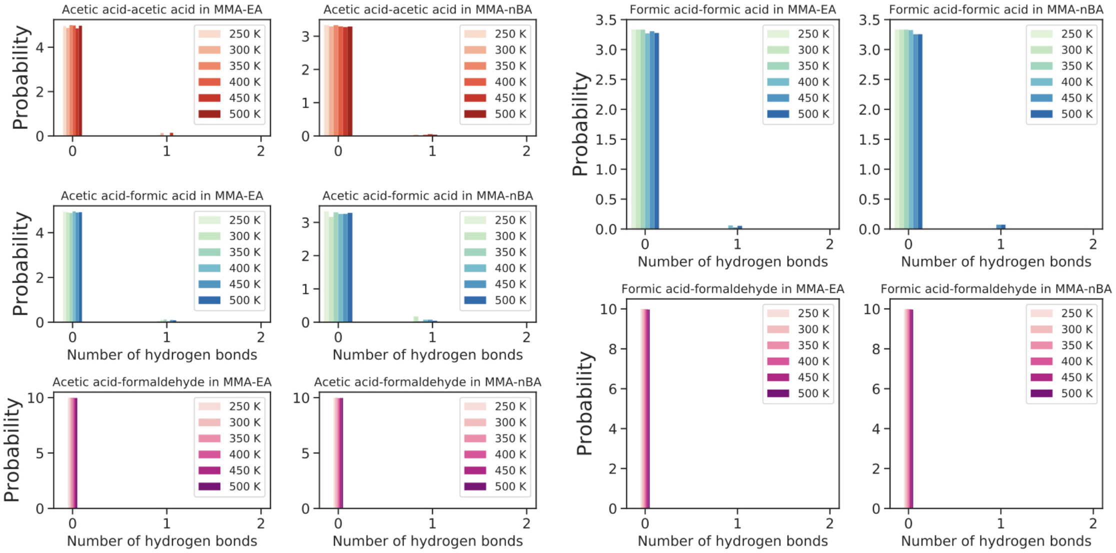

Figure S29: Probability distribution of number of VOC-VOC hydrogen bonds for 6000 ppm concentration.

Interactions of water

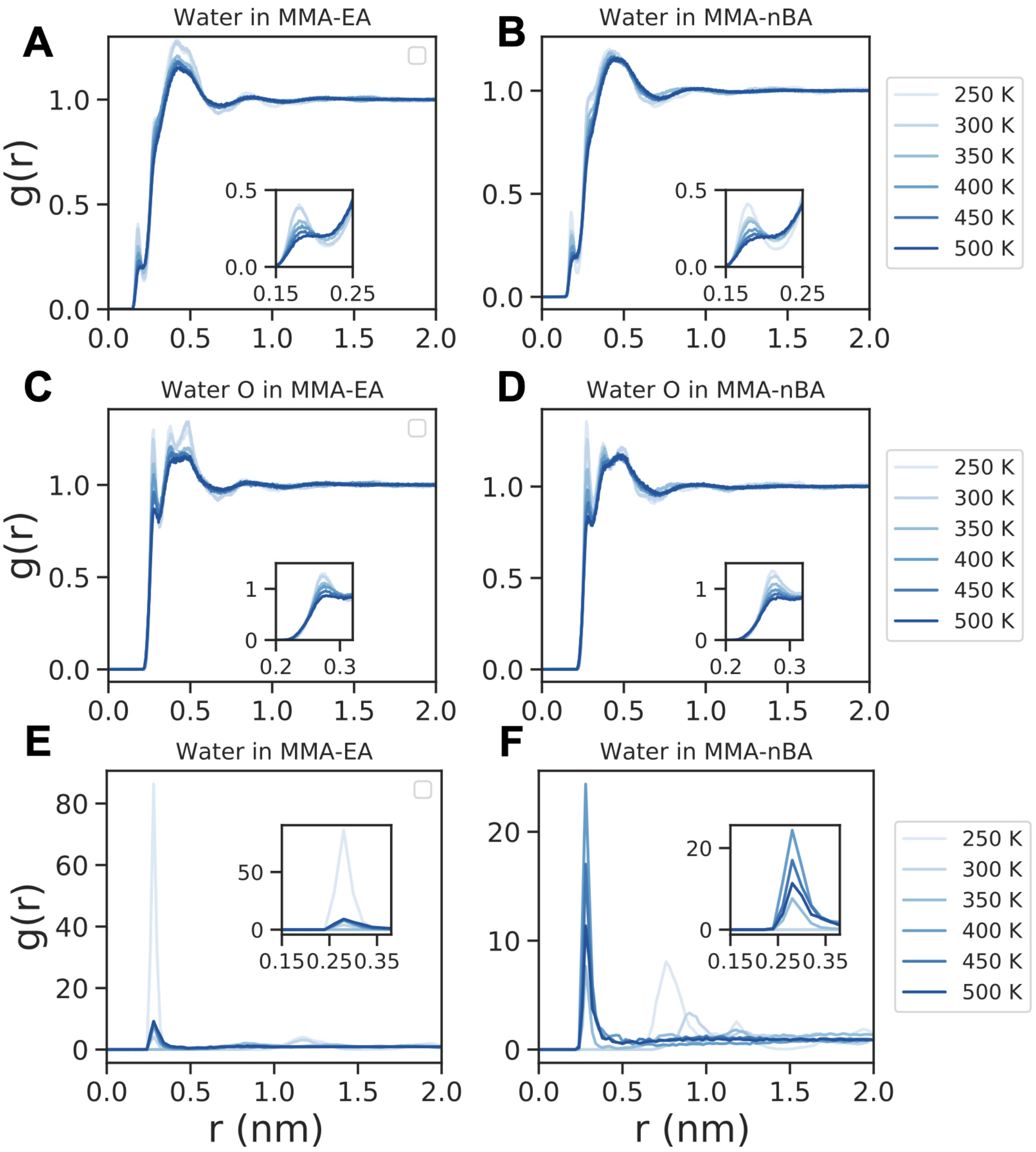

Figure S30: The pair distribution functions, g(r), between (**A,B**) water and copolymer chains (**C,D**) water oxygen and copolymer chains, and (**E,F**) water and other water molecules at different temperatures.

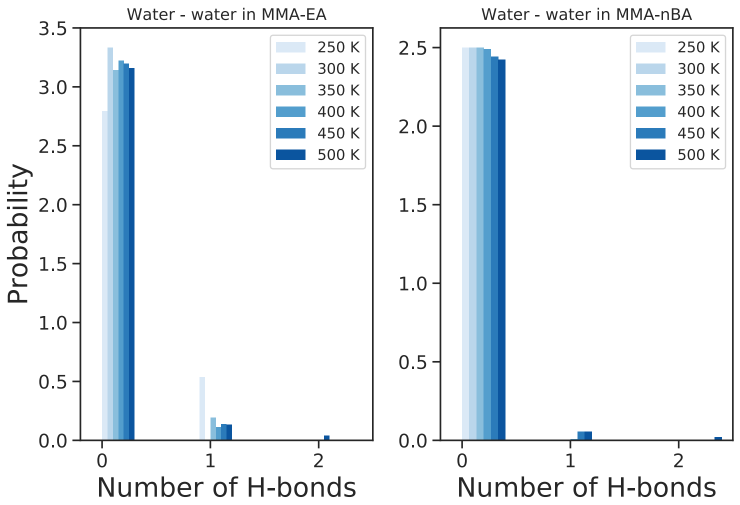

Figure S31: Probability distribution of number of water-water hydrogen bonds.

Structural changes induced by VOCs and water

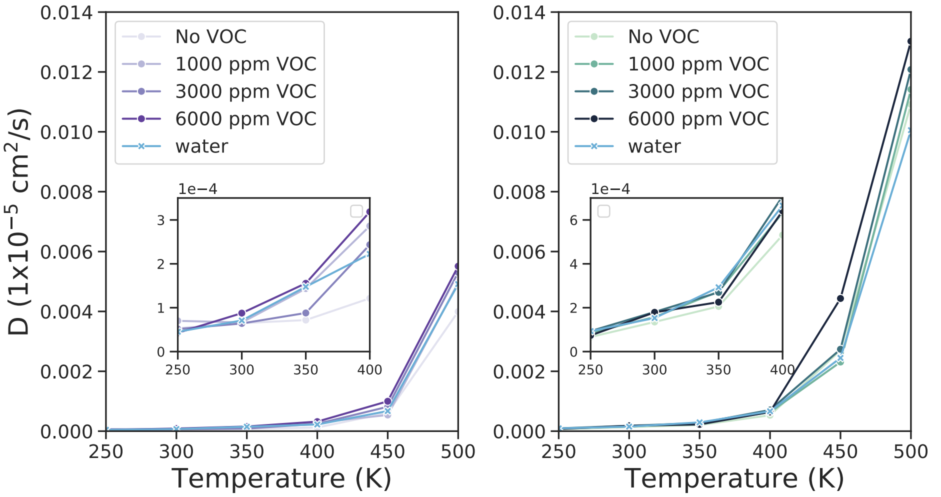

Figure S32: The log of self-diffusion coefficients of copolymer chains with or without pollutants at different temperatures for (**A**) P(MMA-co-EA) and (**B**) P(MMA-co-*n*BA).

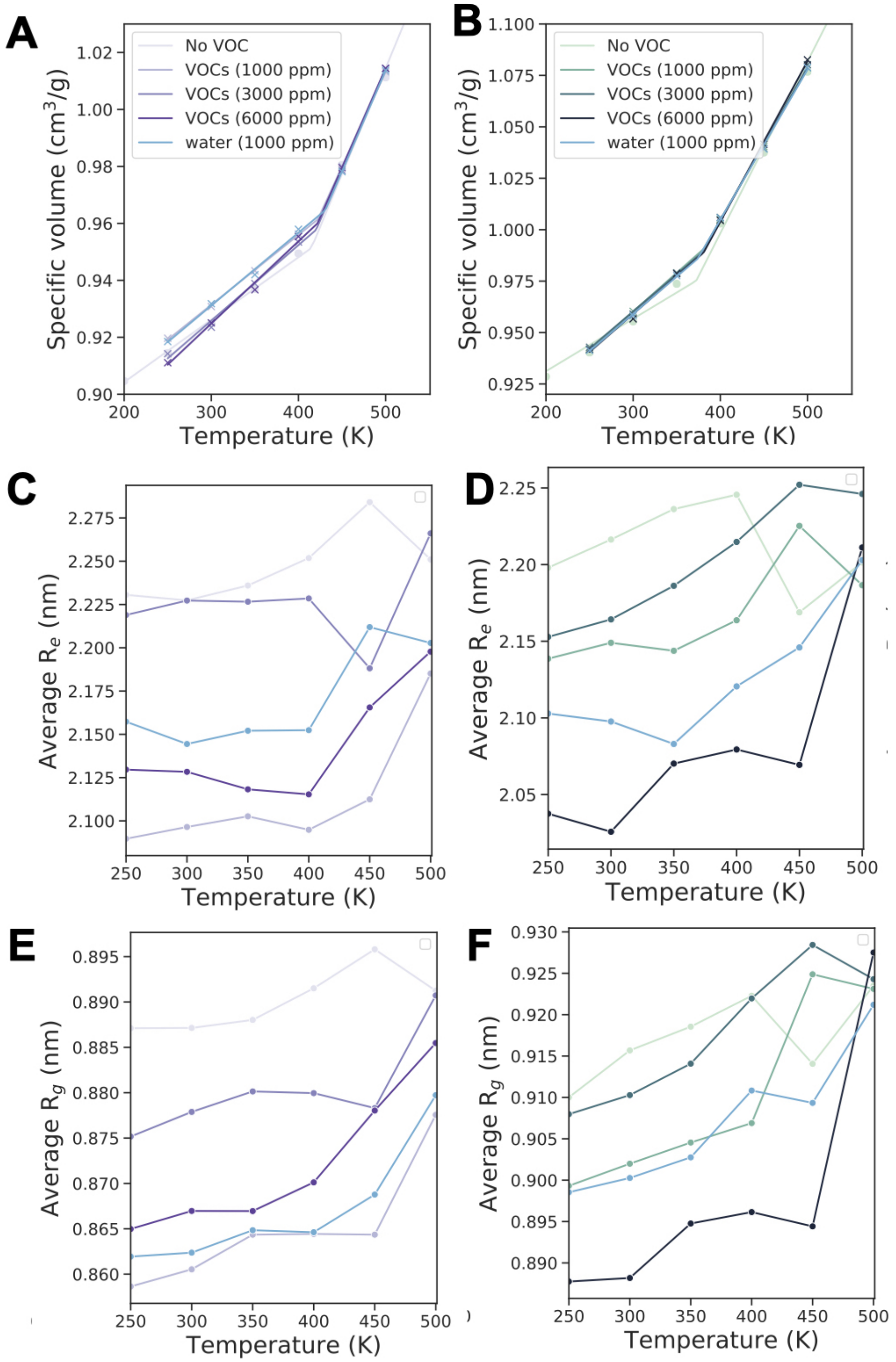

Figure S33: Effect of VOCs and water on (A,B) specific volume and T$_g$, (C,D) average end-to-end distance, and (E,F) average radius of gyration for P(MMA-co-EA) (left panel) and P(MMA-co-nBA) (right panel).

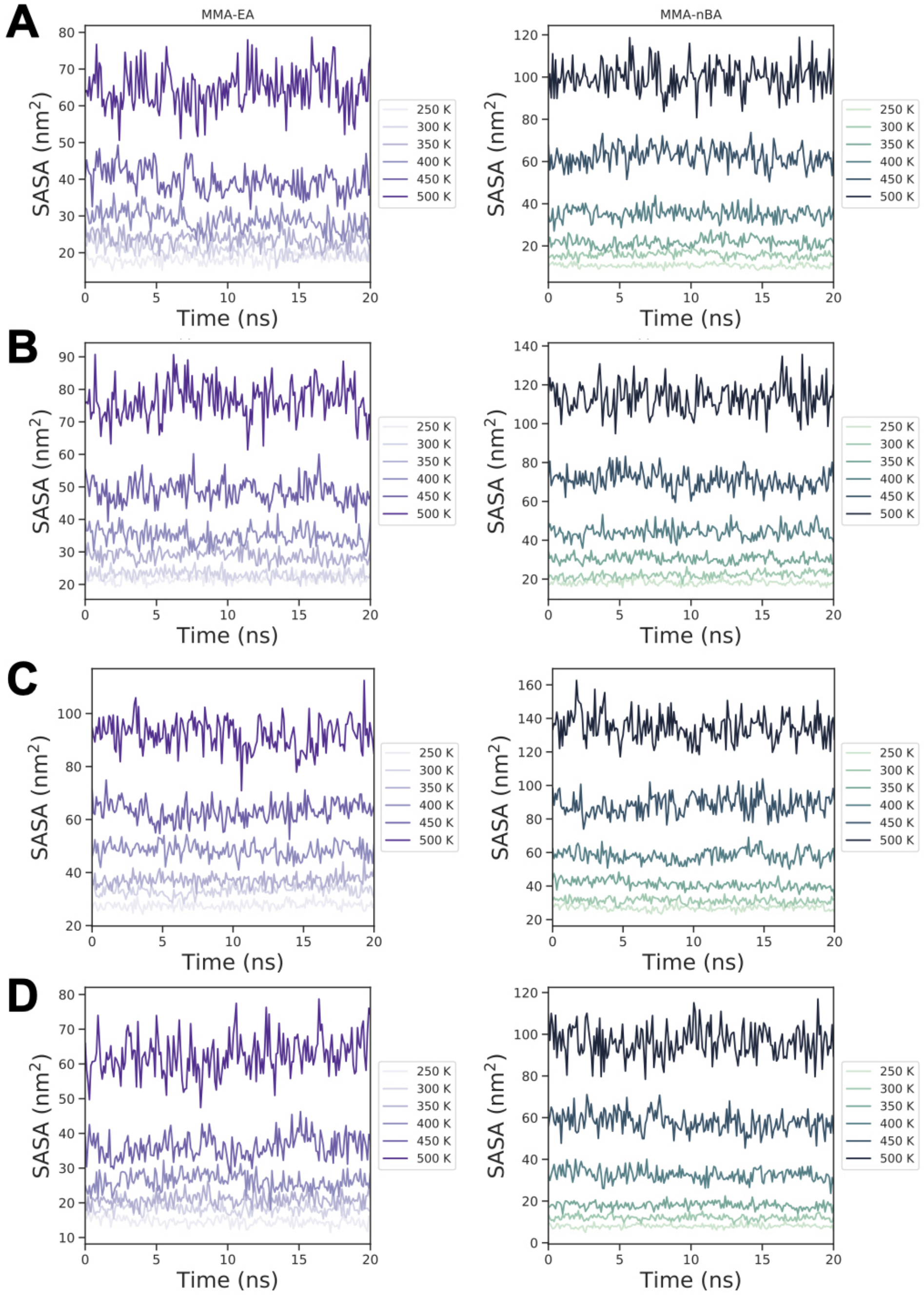

Figure S34: Effect of (A)1000 ppm VOCs, (B) 3000 ppm VOCs, (C) 6000 ppm VOCs and (D) 1000 ppm water on the solvent accessible surface area for P(MMA-co-EA) (left panel) and P(MMA-co-nBA) (right panel).
